# The Reactivity of Azidonitrobenzofuroxans towards 1,3-Dicarbonyl Compounds: Unexpected Formation of Amino Derivative via the Regitz Diazo Transfer and Tautomerism Study

**DOI:** 10.3390/ijms22179646

**Published:** 2021-09-06

**Authors:** Elena Chugunova, Almir Gazizov, Daut Islamov, Alexander Burilov, Alena Tulesinova, Sergey Kharlamov, Victor Syakaev, Vasily Babaev, Nurgali Akylbekov, Nurbol Appazov, Konstantin Usachev, Rakhmetulla Zhapparbergenov

**Affiliations:** 1 Arbuzov Institute of Organic and Physical Chemistry, FRC Kazan Scientific Center, Russian Academy of Sciences, 420088 Kazan, Russia; daut1989@mail.ru (D.I.); burilov_2004@mail.ru (A.B.); khsergey@inbox.ru (S.K.); vsyakaev@iopc.ru (V.S.); babaev-84@mail.ru (V.B.); 2 Laboratory of Plant Infectious Diseases, FRC Kazan Scientific Center of Russian Academy of Sciences, 420111 Kazan, Russia; 3 Institute of Chemical Engineering and Technology, The Kazan National Research Technological University, 420015 Kazan, Russia; tulessinova.a@yandex.ru; 4 Laboratory of Engineering Profile “Physical and Chemical Methods of Analysis”, Korkyt Ata Kyzylorda University, Aitekebie Str. 29A, Kyzylorda 120014, Kazakhstan; nurgali_089@mail.ru (N.A.); ulagat-91@mail.ru (R.Z.); 5 I. Zhakhaev Kazakh Scientific Research Institute of Rice Growing, AbayAvenue 25B, Kyzylorda 120008, Kazakhstan; 6 Kazan Institute of Biochemistry and Biophysics, FRC Kazan Scientific Center of Russian Academy of Sciences, 420111 Kazan, Russia; k.usachev@mail.ru

**Keywords:** benzofuroxan, 1,3-dicarbonyl compounds, Regitz diazo transfer, tautomerism

## Abstract

Herein, we report on the reaction of nitro-substituted azidobenzofuroxans with 1,3-dicarbonyl compounds in basic media. The known reactions of benzofuroxans and azidofuroxans with 1,3-dicarbonyl compounds in the presence of bases are the 1,3-dipolar cycloaddition and the Beirut reaction. In contrast with this, azidonitrobenzofuroxan reacts with 1,3-carbonyl compounds through Regitz diazo transfer, which is the first example of this type of reaction for furoxan derivatives. This difference is seemingly due to the strong electron-withdrawing effect of the superelectrophilic azidonitrobenzofuroxan, which serves as the azido transfer agent rather than 1,3-dipole in this case.

## 1. Introduction

Derivatives of benzo[*c*][1,2,5]oxadiazole 1-oxide (benzofuroxan) have attracted the attention of chemists due to the biological activity exhibited by this class of heterocycle. Benzofuroxans have been proposed as monoamine oxidase inhibitors [1,2] and calcium channel modulators [3,4], and possess vasodilating and cardiotropic [5], antitumor [6,7,8,9], antiparasitic [10,11], anti-tuberculosis [12,13,14], bactericidal [15,16,17], virucidal, sporicidal, and fungicidal activities [18,19]. Moreover, benzofuroxans exhibit anunusual chemical behavior due to the specificity of the electronic structure of the 1,2,5-oxadiazole-*N*-oxide cycle. Benzofuroxans are capable of entering not only substitution reactions [20,21], but also nucleophilic addition and cycloaddition reactions both as a dienophile and as a diene [22,23,24]. Additionally, they are characterized by the phenomenon of tautomerism (benzo[*c*][1,2,5]oxadiazole 1-oxide quickly rearranging into benzo[*c*][1,2,5]oxadiazole 3-oxideviathe open dinitroso form) [25], which often complicates the determination of the structure of asymmetrically substituted benzofuroxans [26] and makes their derivatization a challenging task.

**Scheme 1:**
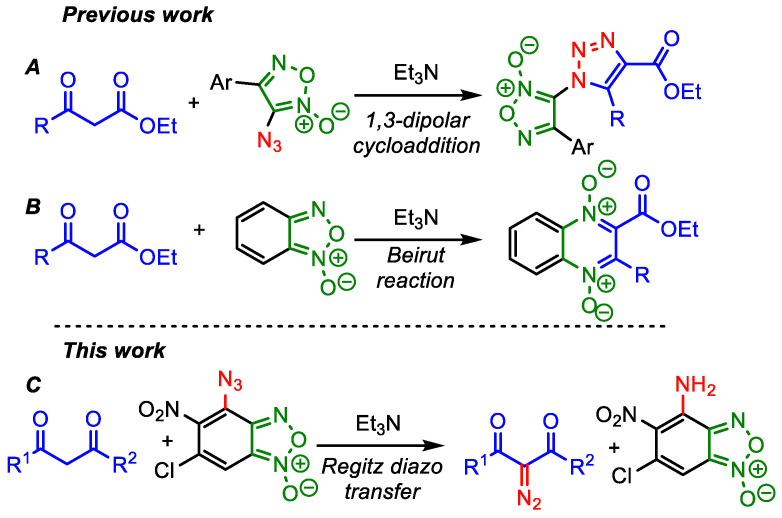
Known reaction modes of the furoxan derivatives with 1,3-dicarbonyl compounds: (**A**) 1,3-dipolar cycloaddition of azidofuroxan derivatives; (**B**) the Beirut reaction of the benzofuroxans; (**C**) the Regitz diazo transfer of the azidobenzofuroxans (this work).

Organic azides are valuable building blocks in synthetic organic chemistry due to the well-known ability of azides to undergo 1,3-dipolar cycloaddition reactions. We speculated that the presence of the azide moiety in the benzofuroxan molecule would allow for easy installation of various biologically relevant fragments via a “click” reaction. Surprisingly, the literature survey revealed that the chemistry of the azidobenzofuroxans is still unexplored. The only reaction of the furoxan-based azide with 1,3-dicarbonyl compounds resulting in the 3+2 cycloaddition in the basic media was reported by Fershtat and coworkers [27] (Scheme 1A). Notably, benzofuroxans are also known to undergo the Beirut reaction under the same conditions [28] (Scheme 1B). Thus, we decided to investigate the possible reaction modes of azidobenzofuroxans with 1,3-dicarbonyl compounds, and herein we report the results of our studies. In contrast with the known examples, superelectrophilic azidobenzofuroxans do not undergo 1,3-dipolar cycloaddition nor the Beirut reaction with 1,3-dicarbonyl compounds. Instead, the Regitz diazo transfer proceeds, which results in the appropriate diazo compound and aminobenzofuroxan (Scheme 1C). Additionally, we explored the obtained tautomerism using NMR and quantum chemistry calculations.

## 2. Results and Discussion

**Figure 1:**
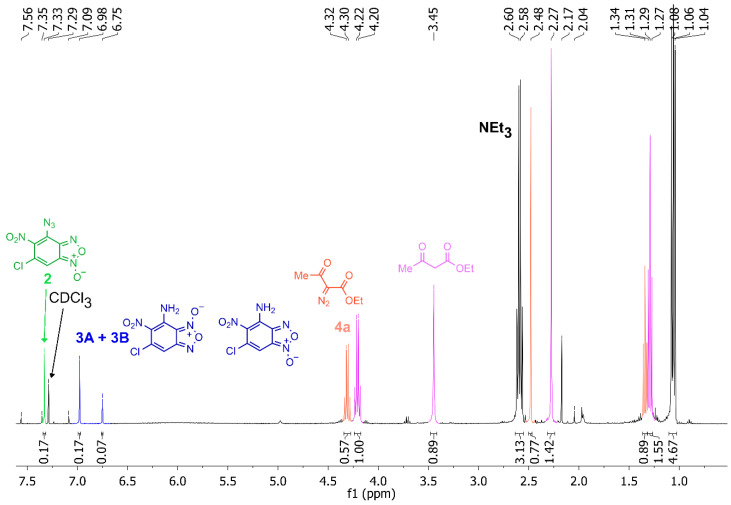
The ^1^H NMR spectra of the reaction mixture of the azidonitrobenzofuroxan **2** with ethylacetoacetate (CDCl_3_, 400 MHz, 303 K).

**Scheme 2:**
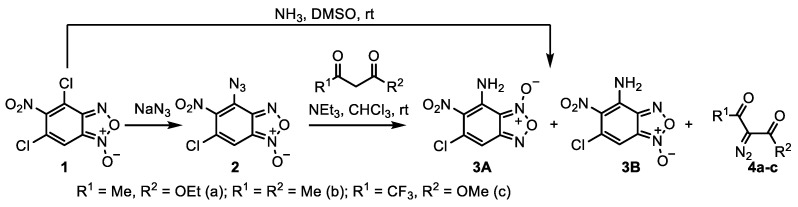
The Regitz diazo transfer of azidobenzofuroxan **2**.

We initiated our studies through the synthesis of azidobenzofuroxan **2** via the S_N_Ar reaction of chloro derivative **1** with sodium azide (Scheme 2). Next, we carried out the reaction of azide **2** with acetoacetic acid ester under the conditions described by Fershtat and coworkers [27]. Surprisingly, the analysis of the mass spectra of the reaction mixture indicated the complete absence of a cycloaddition product. The experiment was repeated in the NMR tube, and careful examination of the ^1^H NMR spectrum revealed signals due to three major products alongside some starting azide 2, starting ester, and triethylamine (Figure 1).

**Figure 2:**
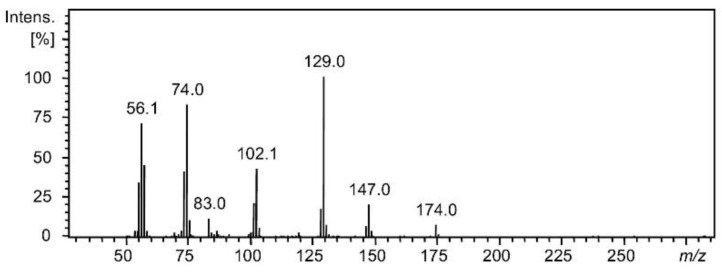
EI mass spectrum of peak of hydrated molecular ion of **4a** [M+H_2_O]^+•^ and the range of the fragmentation peaks obtained from the addition of water to the compound **4a**.

One of these products was identified as ethyl 2-diazo-3-oxobutanoate **4a** by the comparison of the spectrum with the published data [29]. The mass spectrometry data also indicated the presence of the hydrated molecular ion of **4a**. This ion may be formed either from ionisation of the hydrated form of **4a** or through rapid hydration of the ionised form of **4a** during the mass spectrometric experiment. The obtained data do not allow fordistinguishing between these possibilities. In the EI mass spectrum peak of the molecular ion, [M+H_2_O]^+•^ (8%)was observed, as well as the range of the fragmentation peaks (147 [M+H_2_O-C_2_H_3_]^+^ (19%), 129 [M+H_2_O-C_2_H_5_O]^+^ (100%), 102 [M+H_2_O-C_3_H_4_O_2_]^+^ (43%), and 74 [M+H_2_O-C_3_H_4_N_2_O_2_]^+^ (82%); Figure 2).

We speculate that the formation of the diazo compound **4a** may be explained by the diazo transfer reaction described by Regitz [30] (Scheme 2). Based on this, we propose that the singlets in the 6.75–7.34 ppm range (see Figure 1) may be attributed to the tautomeric aminobenzofuroxans **3A** and **3B**, as well as the unreacted starting azidobenzofuroxan **2**.

Similar results were obtained when acetylacetone and trifluoromethyl-3-oxobutanoic acid ester were employed instead of acetoacetic acid ester. Amino derivative **3** was obtained in almost the same yield (*ca* 1–2% difference) alongside the appropriate diazo compound (see Supplementary Materials, Figures S1 and S2 for the spectra). Thus, the replacement of the electron-donating methyl group by the electron-withdrawing trifluoromethyl group, as well as the replacement of the ethoxy fragment by the methyl group, did not affect the reaction course. Taking this into account, we conclude that the reaction outcome was determined by azidofuroxan rather than by the nature of the 1,3-dicarbonyl compound.

This reaction pathway differs from both the 1,3-dipolar cycloaddition of azidofuroxans described by Fershtat and the Beirut reaction of benzofuroxans. Notably, the diazo transfer is the preferred reaction pathway in the case of tosylazide [29], which possesses an electron-withdrawing substituent next to the azide group. At the same time, the more electron-rich phenylazide undergoes cycloaddition with 1,3-dicarbonyl compounds under the same conditions [31,32]. Asnitro-substituted benzofuroxans are known for their superelectrophilic nature [7,21,33,34,35,36], we attribute the difference between azidonitrobenzofuroxan **2** and azidofuroxan derivatives [27] to the strong electron-withdrawing effect of the former.

**Figure 3:**
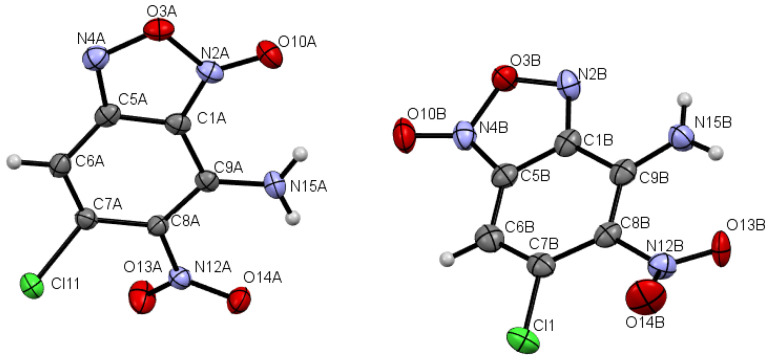
Asymmetric unit of tautomers **3A** and **3B** showing 50% probability thermal ellipsoids. C atoms: grey; N atoms: blue; O atoms: red; Cl atom: green.

Extra evidence was sought to confirm the structure of 3A and 3B. So, aminbenzofuroxan **3** was independently synthesized by the reaction of **1** with excess ammonia in DMSO. The obtained NMR data of the compound appeared to be identical to the signals observed in the NMR of the reaction mixture (Figure 1). Additionally, we were lucky enough to obtain crystals suitable for the X-ray analysis (Figure 3), which also proved the formation of amino derivatives **3A,B**.

The crystals of compound **3** consisted of two tautomers in a ratio of 1:1 (Figure 3). The oxygen atom in one tautomer formed an intramolecular hydrogen bond with the amino group of the other tautomer, while in the second tautomer, the oxygen atom formedan intermolecular hydrogen bond with the amino group. The data on hydrogen bonds are summarized in Table S1, Supplementary Materials. The plane of the nitro group leftthe plane of the benzene ring by 33.2 degrees; apparently this wasdue to the steric factor, asthe neighboring positions in the benzene ring wereoccupied. It should be noted that the tautomers formed planes parallel to 0bc consisting of one type of tautomer (Figure S3, Supplementary Materials).

### Study of the Tautomerism of Benzofuroxans

As mentioned previously, benzofuroxan and its derivatives can undergo tautomerisation, which is believed to proceed through the transient formation of 1,2-dinitrosoarenes (see Supplementary Materials, Figure S4 for the putative mechanism of the tautomerisation). Consequently, the determination of the tautomeric composition of the benzofuroxans is important for the correct determination of their structure. Taking this into account, the study of the tautomerism of compounds used was essential for our research.

First, we investigated the starting chlorobenzofuroxan **1** using various NMR techniques. As mentioned above, one of the main difficulties in studying thetitled compounds using NMR is a lack of a sufficient number of spin markers (protons) in these molecules (vide supra). This strongly complicates «direct» structure elucidation, i.e., using only experimental 2D correlation NMR data. To overcome such problems, anab initio analysis could be applied.

A variety of NMR correlation methods were used to establish the structure of the titled compound. The combination of 2D ^1^H-^13^C HSQC and ^1^H-^15^N/^13^C HMBC correlations enabled directly establishing two heteroaromatic moieties.

**Figure 4:**
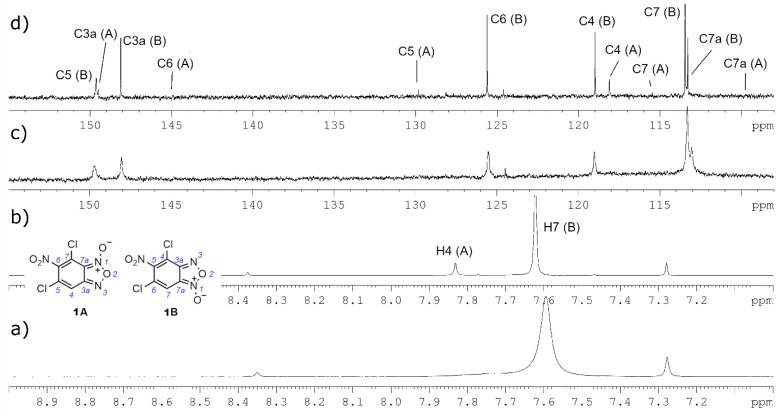
The ^1^H and ^13^C NMR spectra of compound **1**, CDCl_3_: (**a**) ^1^H, 303 K; (**b**) ^1^H, 253 K; (**c**) ^13^C, 303 K; (**d**) ^13^C, 253 K.

The ^1^H NMR spectrum of **1** at 303 K consisted of a broadened (*ca* 20 Hz) signal at 7.6 ppm with a weak broad shoulder on the lowfield side of the signal (Figure 4a). Thus, a chemical exchange wasobserved. DNMR showed that 313 K was a coalescence point, and a high resolution spectrum tookplace for T < 263 K (Figures S5 and S6, Supplementary Materials). The components ratio was *ca* 91:9. Indeed, the theoretical analysis gave a 1.63 kCal/mol energy gap with a preference for the **B** form (Figure 4). Moreover, a barrier of *ca* 18 kCal was estimated.

NMR experiments allowed for establishing C-H connectivities for a part of the molecule. However, the earlier utilized level of theory was used again in order to accomplish the assignment (Table S2, Supplementary Materials). The experimentally identified ^13^C CSs were used as reference points in order to choose an appropriate calculated set. C7a/C3a resonance CS, which was detected experimentally, strongly depends on the tautomer form (ca 110 ppm for **A** (C7a) and 150 ppm for **B** (C3a)) for a trustworthy assignment to bepossible.

The observed dependence of tautomers interconversion rates could probably be explained in terms of electrostatic repulsion. In the case of **1,** the tautomer **A** became less favorable due to an additional chlorine atom on the side of N1, which shifted the equilibrium towards the **B** tautomer.

Additionally, the ^1^H NMR spectra of both chlorobenzofuroxan **1** and aminobenzofuroxan **3**, as well as its *N*-substituted analog **5,** were recorded in various solvents (Figures S5–S23, Table S3, Supplementary Materials) and the ratio of the tautomers is given in the Table 1. As seen from the table, a strong preference for tautomer **B** existed in acetone and chloroform for the chloro derivative **1**. In other solvents, signal broadening due to quick equilibrium was observed. In contrast with this, replacement of the chlorine atom by the amino group increased the amount of the second tautomer significantly and hindered tautomerization, so that signals of both tautomers were clearly distinguished in all solvents. This may be explained by the stabilization of tautomer **A** by the hydrogen bonding between the oxygen atom and amino group. Indeed, the quantum chemistry calculations indicated that in the case of compound **3,** tautomer **A** was *ca* 1 kCal/mol lower in energy than tautomer **B**. The calculated tautomerization barrier was *ca* 23 kCal, which was considerably higher compared with chlorobenzofuroxan **1**, and is in accordance with the experimental observations (Figure S4, Supplementary Materials). Interestingly, in case of its *N*-butyl substituted analog, tautomer **B** again became lower in energy (*ca* 2.5 kCal/mol). This was presumably due to the steric hindrance caused by the *n*-Bu group, which overcame again in energy due to intramolecular hydrogen bonding.

## 3. Conclusions

In conclusion, we discovered that azidonitrobenzofuroxans react with 1,3-carbonyl compounds through the Regitz diazo transfer. This is in sharp contrast with both azidofuroxans and benzofuroxans, which undergo 1,3-dipolar cycloaddition or the Beirut reaction under the same conditions. This difference is presumably explained by the superelectrophilic nature of the azidonitrobenzofuroxan, which serves as an azido transfer agent rather than 1,3-dipole in this case.

## 4. Materials and Methods

### 4.1. Chemistry

The IR spectra were recorded as an emulsion in Vaseline oil (sample concentration 0.25%) on a Tensor 27 (Bruker GmbH, Germany) in the range 400–4000 cm^−1^, which were the most intense absorption bands. Electron ionization (EI) mass spectra were obtained using gas chromatography−mass spectrometry with an Agilent 6890N–5973N (Agilent Technologies, Santa Clara, CA, USA) system at an electron ionization energy of 70 eV, and the temperature of the ion source was250 °C. Nuclear magnetic resonance (NMR) spectra were recorded on Bruker spectrometers AVANCE*III*-500 (BrukerBioSpin, Rheinstetten, Germany) (500.1 MHz for ^1^H, 125.8 MHz for ^13^C) in different solvents at 303 K. Chemical shifts were measured in δ (ppm) with reference to the solvent (δ = 2.10 ppm and 30.5 ppm for (CD_3_)_2_CO for ^1^H and ^13^C NMR, respectively). The pulse programs of the COSY, HSQC, and HMBC experiments were taken from the Bruker software library. Compound **3** obtained using Method 1 (see below) was used in all of the NMR experiments.

Elemental analysis was performed on a CHNS-O Elemental Analyser EuroEA3028-HT-OM (EuroVectorS.p.A., Milan, Italy) with an accuracy ±0.4% for C, H, and N. The melting points were determined in glass capillaries on a Stuart SMP 10 instrument (Keison Products, Chelmsford, UK). The progress of the reactions and the purity of the products were monitored usingTLC on Sorbfil UV-254 plates (Sorbpolimer, Krasnodar, Russia); the chromatograms were developed under UV light.

#### 4.1.1. X-ray Crystallography Data

The data set for the single crystal **3A**/**3B** obtained by Method 1 (see below) was collected on a Rigaku Synergy S instrument (Rigaku Oxford diffraction, Tokyo, Japan) with a HyPix detector and a PhotonJet microfocus X-ray tube using Cu Kα (1.54184 Å) radiation at a low temperature. Images were indexed and integrated using the CrysAlisPro data reduction package. Data were corrected for systematic errors and absorption using the ABSPACK module: numerical absorption correction based on Gaussian integration over a multifaceted crystal model and empirical absorption correction based on spherical harmonics according to the point group symmetry using equivalent reflections. The GRAL module was used for the analysis of the systematic absences and space group determination. The structure was solved withdirect methods using SHELXT [37], and was refined by the full-matrix least-squares on F2 using SHELXL [38]. Non-hydrogen atoms were refined anisotropically. The hydrogen atoms were inserted at the calculated positions and were refined as riding atoms. The figures were generated using the Mercury v4.1 [39] program. Crystals were obtained using the slow evaporation method.

CCDC number 2,099,880 contains the supplementary crystallographic data for this paper. These data can be obtained free of charge via www.ccdc.cam.ac.uk/conts/retrieving.html, accessed on 28 July 2021 (or from the Cambridge Crystallographic Data Centre, 12 Union Road, Cambridge CB2 1EZ, UK; fax: +44 1223-336-033; or deposit@ccdc.cam.uk).

#### 4.1.2. Quantum-Chemical Computations

The GIAO DFT b3lyp/6–31+g(2d,p)//b3lyp/6–31+g(2d,p) basis set was used for geometry optimization and calculation of the NMR parameters, and b3lyp/6–31g(d)//b3lyp/6–31g(d) was used for the SCF energy calculation of thetransition states. Carbons with a chlorine substituent were excluded from the correlation analysis due to a known problem in the theoretical estimations of CS for nuclei of the 3d group of chemical elements [40]. All of the calculations were performed with the Gaussian 16 package [41]. All optimizations were followed by frequency calculations at the same level of theory in order to check that the optimized structures really corresponded to the true minima.

The following compounds were prepared following the literature procedures indicated: 4,6-dichloro-5-nitrobenzo[*c*][1,2,5]oxadiazole 1-oxide **1 [42]** and 4-(butylamino)-6-chloro-5-nitrobenzo[*c*][1,2,5]oxadiazole 1-oxide **5 [21]**.

**The synthesis of the 4-azido-6-chloro-5-nitrobenzo[*c*][1,2,5]oxadiazole 1-oxide 2.**

To a solution of 4,6-dichloro-5-nitrobenzofuroxan **1** (1 mmol) in 5 mL of acetone at room temperature, a solution of sodium azide (1 mmol) in 1 mL of water was added. The reaction mixture was stirred at room temperature for 1h, and the conversion was monitored through TLC analysis (eluent: toluene/ethyl acetate, 2:1). After completion of the reaction, the solvent was evaporated under a vacuum, washed with cold water, and dried in vacuum (0.06 mm Hg) at 40 °C to constant weight. Dark powder, yield 70%; Mp = 60−61 °C; IR (*ν*, cm^–1^): 2127 (N_3_), 1614 (furoxan ring), and 1559 (NO_2_asymm). ^1^H NMR (500 MHz, acetone-*d_6_*): 7.74 (s, 1H). Anal. calcd (%) for C_6_HClN_6_O_4_:C, 28.09; H, 0.39; Cl, 13.82; N, 32.76. Found: C, 28.07; H, 0.41; Cl, 13.84; and N, 32.73.

**The synthesis of the 7-amino-5-chloro-6-nitrobenzo[*c*][1,2,5]oxadiazole 1-oxide 3A and 4-amino-6-chloro-5-nitrobenzo[*c*][1,2,5]oxadiazole 1-oxide 3B.**

Method 1:

To a solution of 4-azido-6-chloro-5-nitrobenzo[*c*][1,2,5]oxadiazole 1-oxide **2** (0.66 mmol) in 5 mL of CH_3_CN or CHCl_3_, triethylamine (0.16 mmol) and ethyl 3-oxobutanoate/pentane-2,4-dione/trifluoromethyl-3-oxobutanoic acid methyl ester (0.66 mmol) were added at room temperature. The reaction was carried out at room temperature and under magnetic stirring, and the conversion was monitored through TLC analysis (eluent: toluene/ethyl acetate, 2:1). The mixture was stirred at room temperature overnight; the solvent was evaporated under vacuum (0.06 mm Hg) at 40 °C to constant weight. The crude product was purified by column chromatography on silica gel (eluent chloroform) to give target compound **3** as the orange powder, with a yield of 56%.

Method 2:

To a solution of 4,6-dichloro-5-nitrobenzofuroxan **1** (1.0 mmol) in 5 mL of DMSO, bubbled dry ammonia (2.0 mmol, the amount was determined by weighing) was added. The reaction mixture was stirred for 1 h at room temperature and was poured into water (30 mL). The obtained solid was filtered off, washed with cold water (50 mL), and dried under a vacuum (0.06 mm Hg) at 40 °C to constant weight.

Orange powder, yield 70%. M.p.: 79–80 °C. IR (*ν*, cm^–1^): 1337 (NO_2_symm), 1558 (NO_2_asymm), 1621 (furoxan ring), and 3336 (NH_2_). MS (EI, 70 eV, Figure S24, Supplementary Materials), *m/z* (I_OTH_ (%)): 214 [M-O]^+^ (75), 184 [M-NO_2_]^+^ (37), 168 [M-O-NO-NH_2_]^+^ (9), and 154 [M-O-2NO]^+^ (42). Anal. calcd (%) for C_6_H_3_ClN_4_O_4_:C, 31.26; H, 1.31; Cl, 15.38; N, 24.30. Found: C, 31.28; H, 1.37; Cl, 15.34; and N, 24.29.

**Acetone-*d*_6_ experiment (the percentage of each tautomer is given in parentheses).**

**Figure 5:**
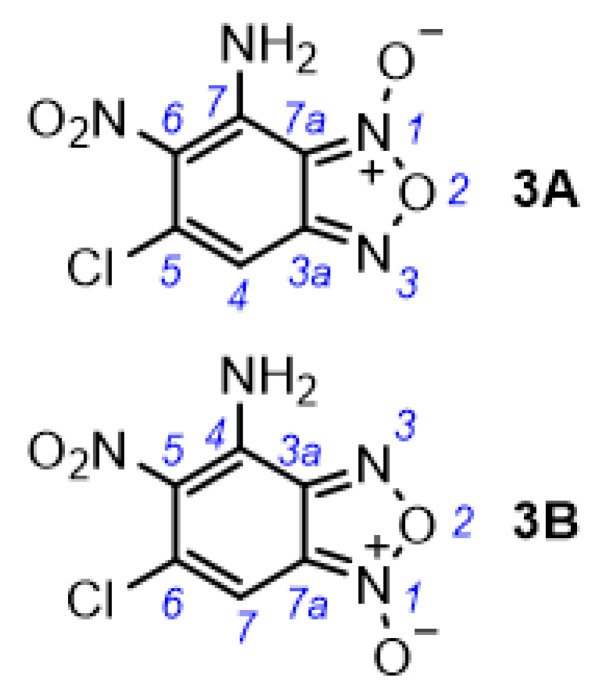
Structures and atoms numbering of tautomers **3A** and **3B**.

7-amino-5-chloro-6-nitrobenzo[*c*][1,2,5]oxadiazole 1-oxide (**3A**, Figure 5) (54%). ^1^H NMR (500 MHz, acetone-*d*_6_): δ 7.16 (s, 1H, H4) and 7.93 (s, 2H, NH_2_). ^13^C{^1^H} NMR (126 MHz, acetone-*d*_6_): δ 151.3 (C7), 137.6 (C5), 133.8 (C3a), 124.2 (C6), 109.0 (C7a), and 105.3 (C4).

4-amino-6-chloro-5-nitrobenzo[*c*][1,2,5]oxadiazole 1-oxide (**3B**, Figure 5) (46%).^1^H NMR (500 MHz, Acetone-*d*_6_): δ 6.91 (s, 1H, H7) and 7.99 (s, 2H, NH_2_). ^13^C{^1^H} NMR (126 MHz, Acetone -*d*_6_): δ 148.0 (C4), 137.7 (C6), 129.0 (C3a), 127.8 (C5), 112.8 (C7a), and 100.3 (C7).

**Benzene-*d_6_* experiment (the percentage of each tautomer is given in parentheses).**

7-amino-5-chloro-6-nitrobenzo[*c*][1,2,5]oxadiazole 1-oxide (**3A)** (89%). ^1^H NMR (500 MHz, benzene-*d_6_*): δ 6.02 (s, 1H, H4) and 6.02 (s, 2H, NH_2_). ^13^C{^1^H} NMR (126 MHz, benzene-*d_6_*): δ 150.5 (C7), 136.3 (C5), 134.3 (C3a), 124.6 (C6), 108.0 (C7a), and 105.6 (C4).

4-amino-6-chloro-5-nitrobenzo[*c*][1,2,5]oxadiazole 1-oxide (**3B**) (11%). As the concentration of the second tautomer wassmall, it practically did not show up in the ^13^C{^1^H} NMR. The chemical shifts of parts of the carbons were determined from the HMBC spectra.^1^H NMR (500 MHz, benzene-*d_6_*): δ 5.77 (s, 1H, H7) and 5.24 (s, 2H, NH_2_). ^13^C{^1^H} NMR (126 MHz, benzene-*d_6_*) δ 146.5 (C4), n/o (C6), 129.4 (C3a), 127.7 (C5), n/o (C7a), and 101.8 (C7).

**Methanol-*d_4_* experiment (the percentage of each tautomer is given in parentheses).**

7-amino-5-chloro-6-nitrobenzo[*c*][1,2,5]oxadiazole 1-oxide (**3A**) (40%). ^1^H NMR (500 MHz, methanol-*d_4_*): δ 7.004 (s, 1H, H4) and n/o (s, 2H, NH_2_). ^13^C{^1^H} NMR (126 MHz, methanol-*d_4_*): δ 151.9 (C7), 137.8 (C5), 134.7 (C3a), 125.0 (C6), 109.4 (C7a), and 105.6 (C4).

4-amino-6-chloro-5-nitrobenzo[*c*][1,2,5]oxadiazole 1-oxide (**3B**) (60%). ^1^H NMR (500 MHz, methanol-*d_4_*): δ 6.761 (s, 1H, H7) and n/o (s, 2H, NH_2_). ^13^C{^1^H} NMR (126 MHz, methanol-*d_4_*) δ 148.6 (C4), 138.4 (C6), 130.1 (C3a), 128.3 (C5), 113.5 (C7a), and 100.2 (C7).

**Crystal Data** for C_6_H_3_ClN_4_O_4_ (*M* = 230.57 g/mol): orthorhombic, space group P2_1_2_1_2_1_ (no. 19), *a* = 5.8860(2) Å, *b* = 11.2467(3) Å, *c* = 24.7322(7) Å, *V* = 1637.22(8) Å^3^, *Z* = 8, *T* = 100.0(4) K, μ(Cu Kα) = 4.246 mm^−1^, *Dcalc* = 1.871 g/cm^3^, 8428 reflections measured (7.148° ≤ 2Θ ≤ 153.52°), 3305 unique (*R*_int_ = 0.0410, R_sigma_ = 0.0393), which were used in all calculations. The final *R*_1_ was 0.0439 (I > 2σ(I)) and *wR*_2_ was 0.1199 (all data).

## Figures and Tables

**Table 1:** Tautomeric ratio for the benzofuroxans **1**, **3,** and **5** in various solvents.

Compound	Solvent			
	CDCl_3_	Acetone-*d_6_*	Methanol-*d_4_*	Benzene-*d_6_*
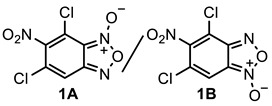	9:91 ^a^	1:140 ^b^	*n*/o	*n*/o
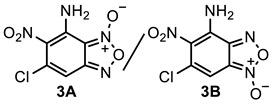	2:1 ^b^	1.2:1 ^b^	1:1.5 ^b^	8:1 ^b^
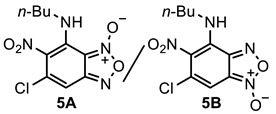	1:34 ^b^	1:34 ^b^	1:32 ^b^	1:19 ^b^

^a^ According to the^1^H NMR data, the spectra were recorded at 253 K; ^b^ the spectra were recorded at 303 K; *n*/o: tautomers not observed due to quick equilibrium.

## Data Availability

The data presented in this study are contained within the article or in Supplementary Materials, or are available upon request from the corresponding author Elena Chugunova.

## Supplementary Materials

The following are available online at https://www.mdpi.com/article/10.3390/ijms22179646/s1.

## References

[1] Severina I.S., Axenova L.N., Veselovsky A.V., Pyatakova N.V., Buneeva O.A., Ivanov A.S., Medvedev A.E. (2003). Nonselective inhibition of monoamine oxidases A and B by activators of soluble guanylate cyclase. Biochemistry.

[2] Bolt A.G., Ghosh P.B., Sleigh M.J. (1974). Benzo-2,1,5-oxadiazoles—A novel class of heterocyclic monoamine oxidase inhibitors. Biochem. Pharmacol..

[3] Gasco A.M., Ermondi G., Fruttero R., Gasco A. (1996). Benzofurazanyl- and benzofuroxanyl-l,4-dihydropyridines: Synthesis, structure and calcium entry blocker activity. Eur. J. Med. Chem..

[4] Visentin S., Amiel P., Fruttero R., Boschi D., Roussel C., Giusta L., Carbone E., Gasco A. (1999). Synthesis and Voltage-Clamp Studies of Methyl Racemates and Enantiomers and of Their Benzofuroxanyl Analogues. J. Med. Chem..

[5] Cerecetto H., Porcal W. (2005). Pharmacological properties of furoxans and benzofuroxans: Recent developments. Mini Rev. Med. Chem..

[6] Farias C.F., Massaoka M.H., Girola N., Azevedo R.A., Ferreira A.K., Jorge S.D., Tavares L.C., Figueiredo C.R., Travassos L.R. (2015). Benzofuroxan derivatives N-Br and N-I induce intrinsic apoptosis in melanoma cells by regulating AKT/BIM signaling and display anti metastatic activity in vivo. BMC Cancer.

[7] Smolobochkin A., Gazizov A., Sazykina M., Akylbekov N., Chugunova E., Sazykin I., Gildebrant A., Voronina J., Burilov A., Karchava S. (2019). Synthesis of Novel 2-(Het)arylpyrrolidine Derivatives and Evaluation of Their Anticancer and Anti-Biofilm Activity. Molecules.

[8] Chugunova E., Micheletti G., Telese D., Boga C., Islamov D., Usachev K., Burilov A., Tulesinova A., Voloshina A., Lyubina A. (2021). Novel Hybrid Compounds Containing Benzofuroxan and Aminothiazole Scaffolds: Synthesis and Evaluation of Their Anticancer Activity. Int. J. Mol. Sci..

[9] Chugunova E., Gazizov A., Sazykina M., Akylbekov N., Gildebrant A., Sazykin I., Burilov A., Appazov N., Karchava S., Klimova M. (2020). Design of Novel 4-Aminobenzofuroxans and Evaluation of Their Antimicrobial and Anticancer Activity. Int. J. Mol. Sci..

[10] Porcal W., Hernández P., Boiani L., Boiani M., Ferreira A., Chidichimo A., Cazzulo J.J., Olea-Azar C., González M., Cerecetto H. (2008). New trypanocidal hybrid compounds from the association of hydrazone moieties and benzofuroxanheterocycle. Bioorg. Med. Chem..

[11] Dos Santos Petry L., Pillar Mayer J.C., de Giacommeti M., Teixeira de Oliveira D., RaziaGarzon L., Martiele Engelmann A., Magalhães de Matos A.F.I., DellaméaBaldissera M., Dornelles L., Melazzo de Andrade C. (2021). In vitro and in vivo trypanocidal activity of a benzofuroxan derivative against Trypanosomacruzi. Exp. Parasitol..

[12] Fernandes G.F.S., Campos D.L., Da Silva I.C., Prates J.L.B., Pavan A.R., Pavan F.R., Dos Santos J.L. (2021). Benzofuroxan Derivatives as Potent Agents against Multidrug-Resistant *Mycobacterium tuberculosis*. ChemMedChem.

[13] Dos Santos Fernandes G.F., de Souza P.C., Marino L.B., Chegaev K., Guglielmo S., Lazzarato L., Fruttero R., Chung M.C., Pavan F.R., dos Santos J.L. (2016). Synthesis and biological activity of furoxan derivatives against *Mycobacterium tuberculosis*. Eur. J. Med. Chem..

[14] Ferreira A.K., Pasqualoto K.F.M., Kruyt F.A.E., Palace-Berl F., Azevedo R.A., Turra K.M., Rodrigues C.P., Ferreira A.C.F., Salomon M.A.C., de Sa Junior P.L. (2016). BFD-22 a new potential inhibitor of BRAF inhibits the metastasis of B16F10 melanoma cells and simultaneously increased the tumor immunogenicity. Toxicol. Appl. Pharmacol..

[15] Jorge S.D., Palace-Berl F., Masunari A., Cechinel C.A., Ishii M., Pasqualoto K.F.M., Tavares L.C. (2011). Novel benzofuroxan derivatives against multidrug-resistant Staphylococcus aureus strains: Design using Topliss’ decision tree, synthesis and biological assay. Bioorg. Med. Chem..

[16] Galkina I.V., Takhautdinova G.L., Tudrii E.V., Yusupova L.M., Falyakhov I.F. (2013). Synthesis, Structure, and Antibacterial Activity of Aminobenzofuroxan and Aminobenzofurazan. J. Org. Chem..

[17] Galkina I.V., Tudriy E.V., Bakhtiyarova Y.V., Usupova L.M., Shulaeva M.P., Pozdeev O.K., Egorova S.N., Galkin V.I. (2014). Synthesis and Antimicrobial Activity of Bis-4,6-sulfonamidated 5,7-Dinitrobenzofuroxans. J. Chem..

[18] Wang L., Li C., Zhang Y., Qiao C. (2013). Synthesis and Biological Evaluation of Benzofuroxan Derivatives as Fungicides against Phytopathogenic Fungi. J. Agric. Food Chem..

[19] Wang L.L., Zhang Y.-Y., Wang L.L., Liu F., Cao L., Yang J., Qiao C., Ye Y. (2014). Benzofurazan derivatives as antifungal agents against phytopathogenic fungi. Eur. J. Med. Chem..

[20] Kotovskaya S.K., Romanova S.A., Charushin V.N., Kodess M.I., Chupakhin O.N. (2004). 5(6)-Fluoro-6(5)-R-benzofuroxans: Synthesis and NMR ^1^H, ^13^C and ^19^F studies. J. Fluor. Chem..

[21] Chugunova E., Frenna V., Consiglio G., Micheletti G., Boga C., Akylbekov N., Burilov A., Spinelli D. (2020). On the Nucleophilic Reactivity of 4,6-Dichloro-5-nitrobenzofuroxan with Some Aliphatic and Aromatic Amines: Selective Nucleophilic Substitution. J. Org. Chem..

[22] Sepulcri P., Hallé J.C., Goumont R., Riou D., Terrier F., Halle J.C., Goumont R., Riou D., Terrier F. (1999). Competitive and consecutive inverse and normal electron demand cycloadditions in the reaction of 4,6-dinitrobenzofuroxan with cyclopentadiene. J. Org. Chem..

[23] Sepulcri P., Goumont R., Hallé J.C., Riou D., Terrier F. (2000). New reactivity patterns in the Diels-Alder reactivity of nitrobenzofuroxans. J. Chem. Soc. Perkin Trans. 2.

[24] Vichard D., Hallé J.-C., Huguet B., Pouet M.-J., Terrier F., Vichard D., Huguet B., Riou D. (1998). A new feature in the chemistry of nitrobenzofuroxans: Ambident reactivity in Diels–Alder condensations. Chem. Commun..

[25] Katritzky A.R., Gordeev M.F. (1993). Heterocyclic rearrangements of benzofuroxans and related compounds. Heterocycles.

[26] Green A.G., Rowe F.M. (1913). The constitution of oxadiazole oxides (furazan oxides or dioxime peroxides). J. Chem. Soc. Trans..

[27] Kulikov A.S., Larin A.A., Fershtat L.L., Anikina L.V., Pukhov S.A., Klochkov S.G., Struchkova M.I., Romanova A.A., Ananyev I.V., Makhova N.N. (2017). Synthesis, structural characterization and cytotoxic activity of heterocyclic compounds containing the furoxan ring. Arkivoc.

[28] Jaso A., Zarranz B., Aldana I., Monge A. (2005). Synthesis of New Quinoxaline-2-carboxylate 1,4-Dioxide Derivatives as Anti-*Mycobacterium tuberculosis* Agents. J. Med. Chem..

[29] Barlind J.G., Bauer U.A., Birch A.M., Birtles S., Buckett L.K., Butlin R.J., Davies R.D.M., Eriksson J.W., Hammond C.D., Hovland R. (2012). Design and Optimization of Pyrazinecarboxamide-Based Inhibitors of DiacylglycerolAcyltransferase 1 (DGAT1) Leading to a Clinical Candidate DimethylpyrazinecarboxamidePhenylcyclohexylacetic Acid (AZD7687). J. Med. Chem..

[30] Regitz M., Maas G. (1986). Diazo Group Transfer. Diazo Compounds.

[31] Wang L., Xu S., Liu X., Chen X., Xiong H., Hou S., Zou W., Tang Q., Zheng P., Zhu W. (2018). Discovery of thinopyrimidine-triazole conjugates as c-Met targeting and apoptosis inducing agents. Bioorg. Chem..

[32] L’Abbe G., Dehaen W. (1988). Synthesis and thermal rearrangement of 5-diazomethyl-1,2,3-triazoles. Tetrahedron.

[33] Chugunova E.A., Sazykina M.A., Gibadullina E.M., Burilov A.R., Sazykin I.S., Chistyakov V.A., Timasheva R.E., Krivolapov D.B., Goumont R. (2013). Synthesis, genotoxicity and UV-protective activity of new benzofuroxans substituted by aromatic amines. Lett. Drug Des. Discov..

[34] Gibadullina E.M., Chugunova E.A., Mironova E.V., Krivolapov D.B., Burilov A.R., Yusupova L.M., Pudovik M.A. (2012). Reaction of 4,6-Dichloro-5-nitrobenzofuroxan with aromatic amines and nitrogen-containing heterocycles. Chem. Heterocycl. Compd..

[35] Serkov I.V., Chugunova E.A., Burilov A.R., Voloshina A.D., Kulik N.V., Zobov V.V., Proshin A.N. (2013). Benzofuroxans containing NO-generating fragment. Russ. J. Gen. Chem..

[36] Chugunova E.A., Mukhamatdinova R.E., Burilov A.R. (2015). Reactions of benzofuroxans with aminoalkyltriphenylphosphonium bromides. Russ. J. Gen. Chem..

[37] Sheldrick G.M. (2015). SHELXT—Integrated space-group and crystal-structure determination. Acta Crystallogr. Sect. A Found. Crystallogr..

[38] Sheldrick G.M. (2008). A short history of SHELX. Acta Crystallogr. Sect. A Found. Crystallogr..

[39] Macrae C.F., Edgington P.R., McCabe P., Pidcock E., Shields G.P., Taylor R., Towler M., van de Streek J. (2006). Mercury: Visualization and analysis of crystal structures. J. Appl. Crystallogr..

[40] Balandina A., Kalinin A., Mamedov V., Figadère B., Latypov S. (2005). Structure-NMR chemical shift relationships for novel functionalized derivatives of quinoxalines. Magn. Reson. Chem..

[41] Frisch M.J., Trucks G.W., Schlegel H.B., Scuseria G.E., Robb M.A., Cheeseman J.R., Scalmani G., Barone V., Petersson G.A., Nakatsuji H. (2016). Gaussian 16, Revision, C.01 2016.

[42] Yusupova L.M., Molodykh Z.V., Buzykin B.I., Falyakhov I.F., Anisimova N.N., Sharnin G.P., Bulidorov V.V., Sviridov S.I., Levinson F.S. (1995). 4- or 6-Nitro-5,7-Dichlorobenzofuroxan Having Fungicidal Activity. RU Patent.

