# Supplementary material for: The Reactivity of Azidonitrobenzofuroxans towards 1,3-Dicarbonyl Compounds: Unexpected Formation of Amino Derivative via the Regitz Diazo Transfer and Tautomerism Study
Source: Int J Mol Sci. 2021 Sep 6;22(17):9646. doi: 10.3390/ijms22179646 (PMC8431794; doi:10.3390/ijms22179646)
Supplement: Supplementary file 1 [file ijms-22-09646-s001.zip › ijms-1340168-supplementary.pdf]

SUPPLEMENTARY MATERIALS

The Reactivity of Azidonitrobenzofuroxans Towards 1,3-Dicarbonyl Compounds: Unexpected formation of Amino Derivative via the Regitz Diazo Transfer and Tautomerism Study

Elena Chugunova [1,2,*], Almir Gazizov [1,2*], Daut Islamov [1], Alexander Burilov [1,2], Alena Tulesinova [3], Sergey Kharlamov [1], Victor Syakaev [1], Vasily Babaev [1], Nurgali Akylbekov [4], Nurbol Appazov [4,5*], Konstantin Usachev [6], Rakhmetulla Zhapparbergenov [4]

1 Arbuzov Institute of Organic and Physical Chemistry, FRC Kazan Scientific Center, Russian Academy of Sciences, Kazan 420088, Russia; daut1989@mail.ru (D.I.); burilov_2004@mail.ru (A.B.); khsergey@inbox.ru (S.Kh.); vsyakaev@iopc.ru (V.S.); babaev-84@mail.ru (V.B.)
2 Laboratory of Plant Infectious Diseases, FRC Kazan Scientific Center of Russian Academy of Sciences, Kazan 420111, Russia
3 Institute of Chemical Engineering and Technology, The Kazan National Research Technological University, Kazan 420015, Russia; tulessinova.a@yandex.ru
4 Laboratory of engineering profile "Physical and chemical methods of analysis", Korkyt Ata Kyzylorda University, Aiteke bie str., 29A, Kyzylorda 120014, The Republic of Kazakhstan; nurgali_089@mail.ru (N.A.), ulagat-91@mail.ru (R.Zh.)
5 I. Zhakhaev Kazakh Scientific Research Institute of Rice Growing, Abay avenue, 25B, Kyzylorda 120008, Kazakhstan;
6 Kazan Institute of Biochemistry and Biophysics, FRC Kazan Scientific Center of Russian Academy of Sciences, Kazan 420111, Russia; k.usachev@mail.ru
* chugunova.e.a@gmail.com (E.C.); agazizov@iopc.ru (A.G.); nurasar.82@mail.ru (N.A.); Tel.: +7-843-272-7324 (E.C., A.G.), +7-724-223-1041 (N.A.)

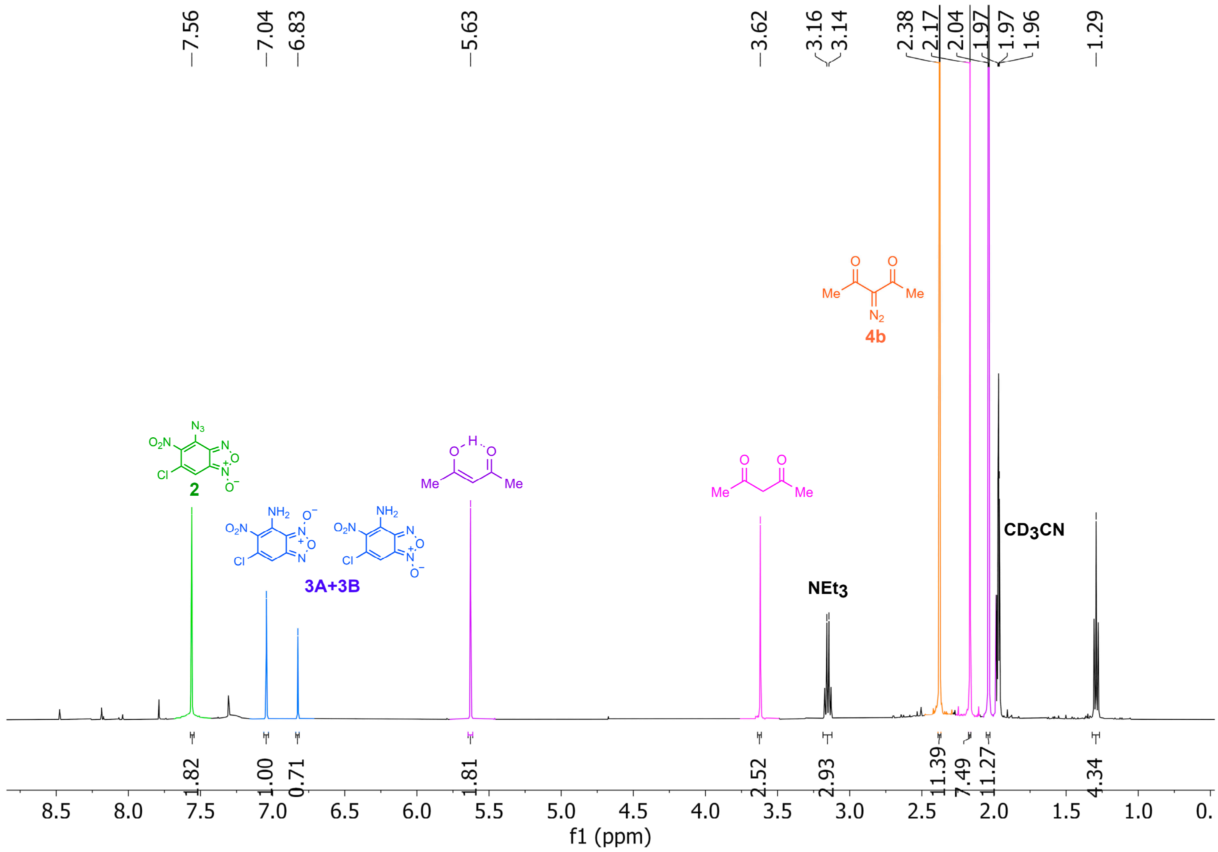

Figure S1. The ^{1}H NMR spectra of the reaction mixture of the azidobenzofuroxan **2** with acetylacetone (CD_3CN, 400 MHz, 303K).

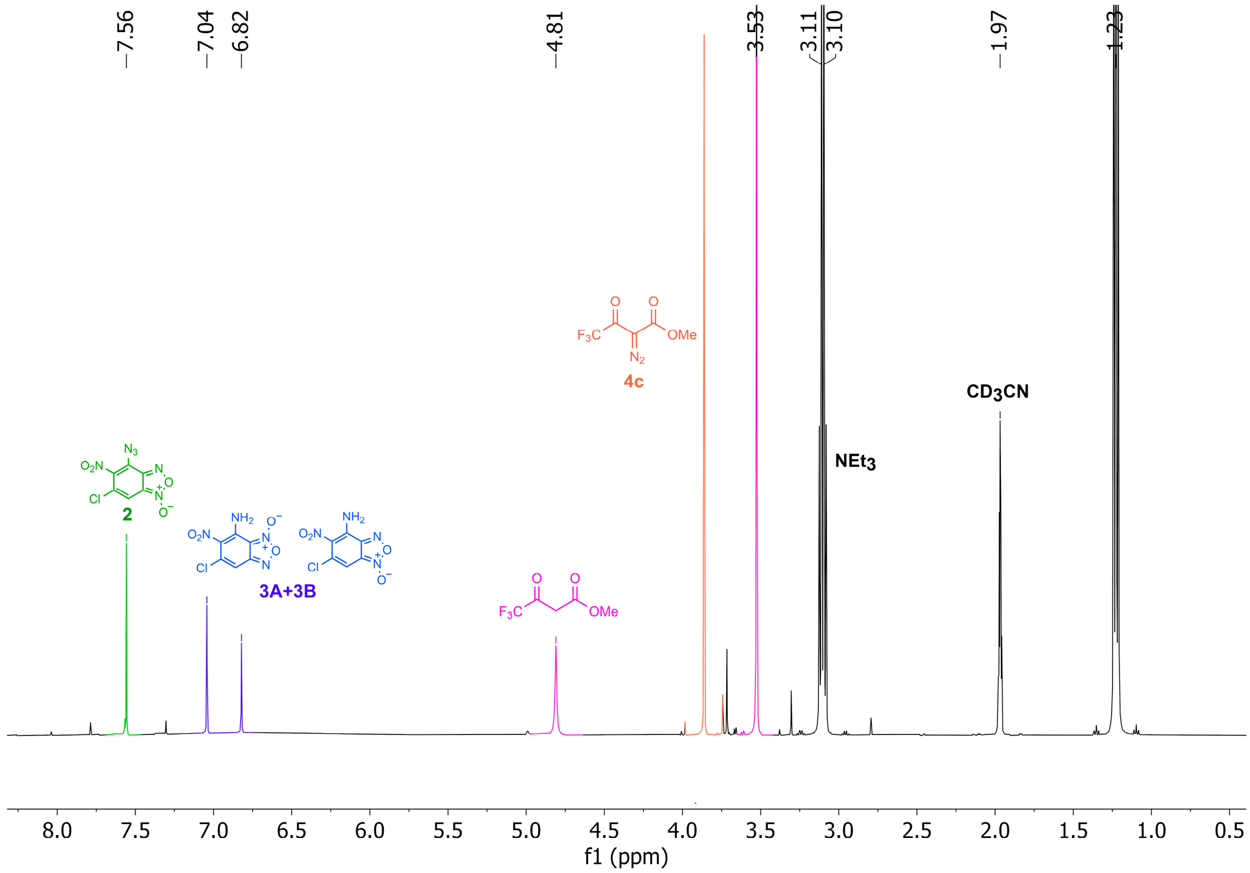

Figure S2. The ^{1}H NMR spectra of the reaction mixture of the azidobenzofuroxan **2** with trifluoromethyl-3-oxobutanoic acid ester (CD_3CN, 400 MHz, 303K).

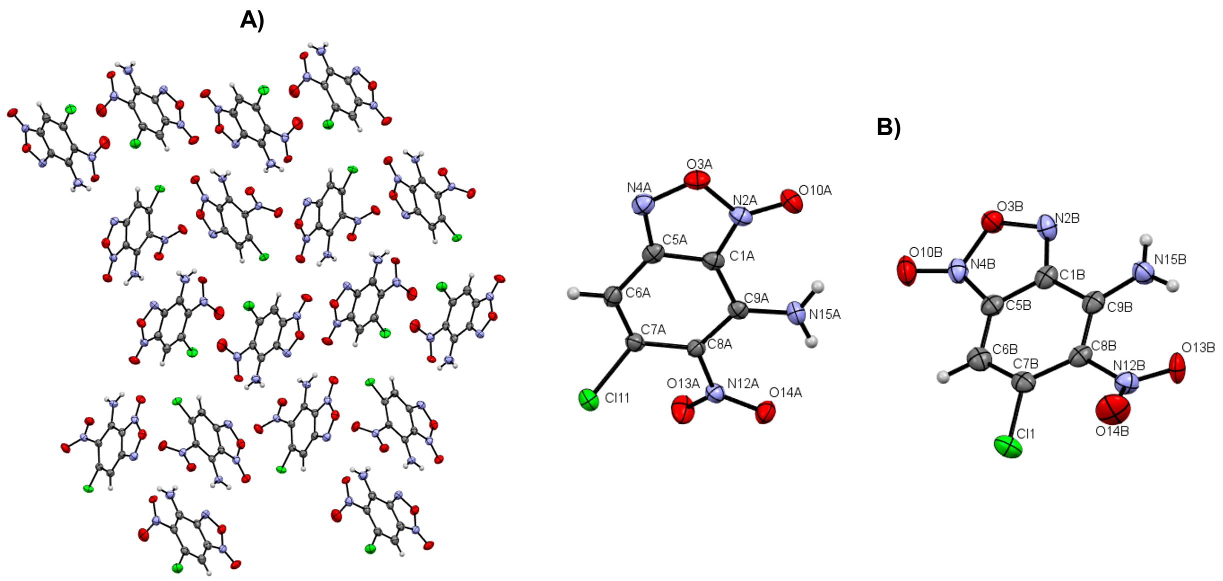

Figure S3. A) Crystal packing of **3** view along *a* axes; B) Asymmetric unit of tautomers **3A** and **3B** showing 50% probability thermal ellipsoids. C atoms –grey, N atoms – blue, O atoms – red, Cl atom - green.

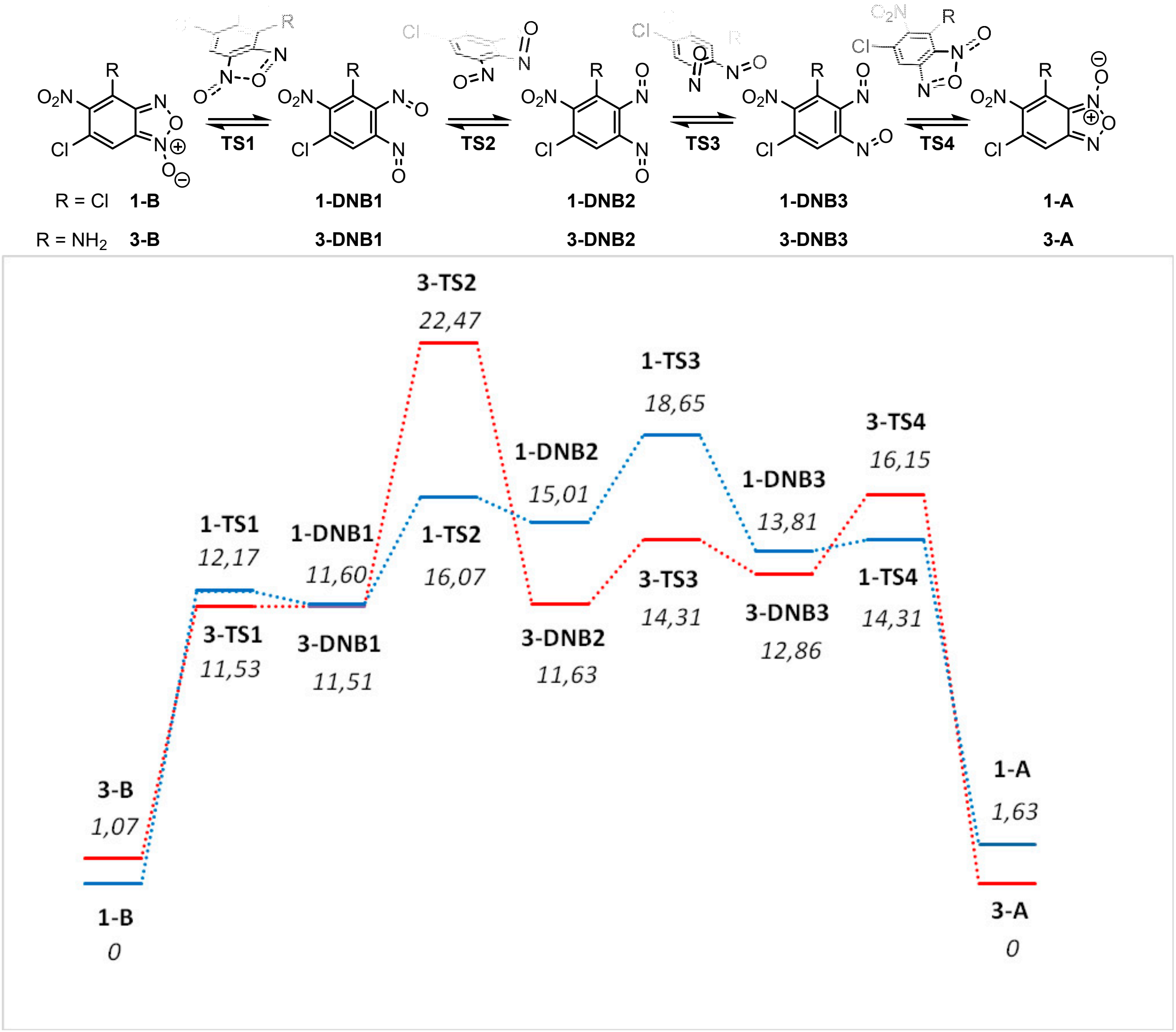

Figure S4. Isomerization mechanism of benzofuroxans **1**,**3** and energy diagram (kCal/mol) as obtained from quantum chemistry calculations (B3LYP/6-31+g(2d,p), Gaussian 16)

Table S1. Hydrogen-bond geometry in crystals of the compound **3**.

D—H•••A	D—H, Å	H•••A, Å	D•••A, Å	D—H•••A, °
**N15A—H15C•••O14A**	0.86	2.06	2.638 (5)	123.9
**N15A—H15D•••O10B**	0.86	2.10	2.800 (4)	138.3
**N15B—H15A•••O13B**	0.86	1.98	2.543 (7)	121.9
**N15B—H15A•••O13C**	0.86	2.05	2.636 (13)	124.9

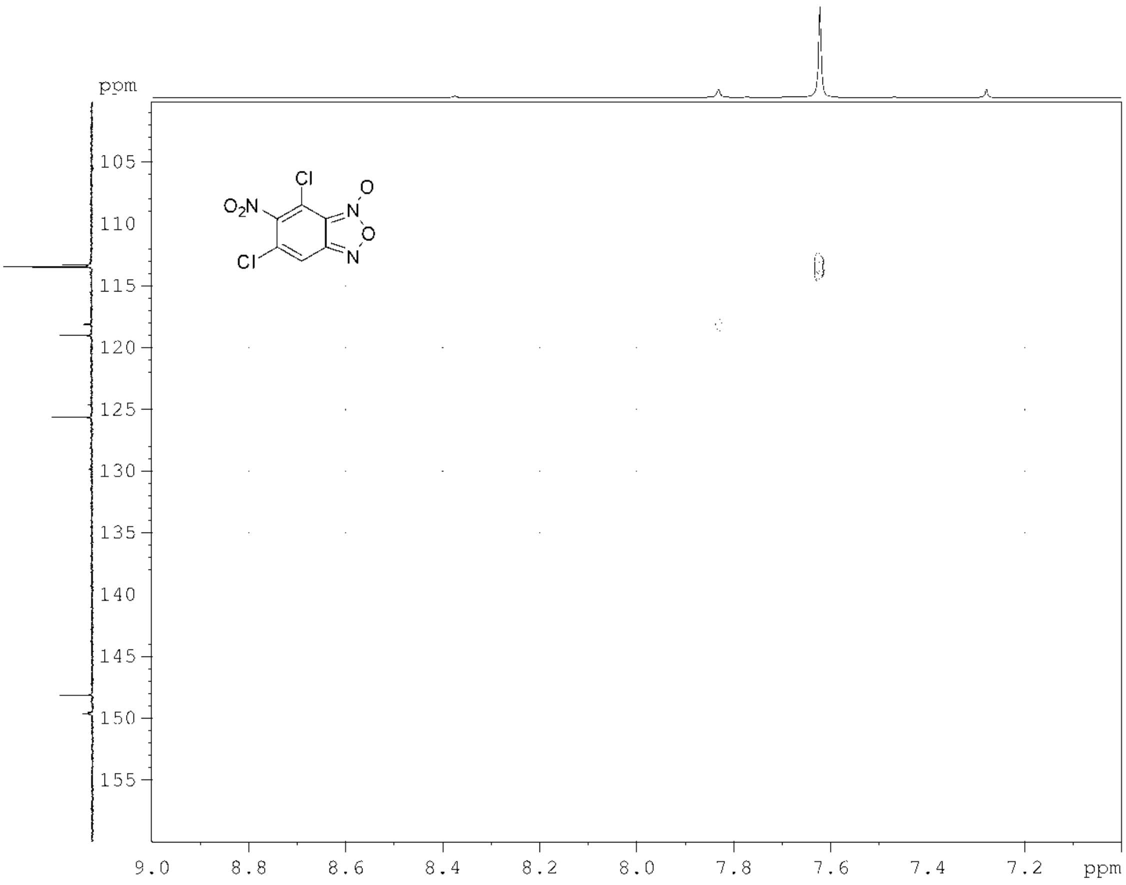

Figure S5. ^{1}H-^{13}C HSQC NMR spectrum of **1**, $CDCl_3$, 253K.

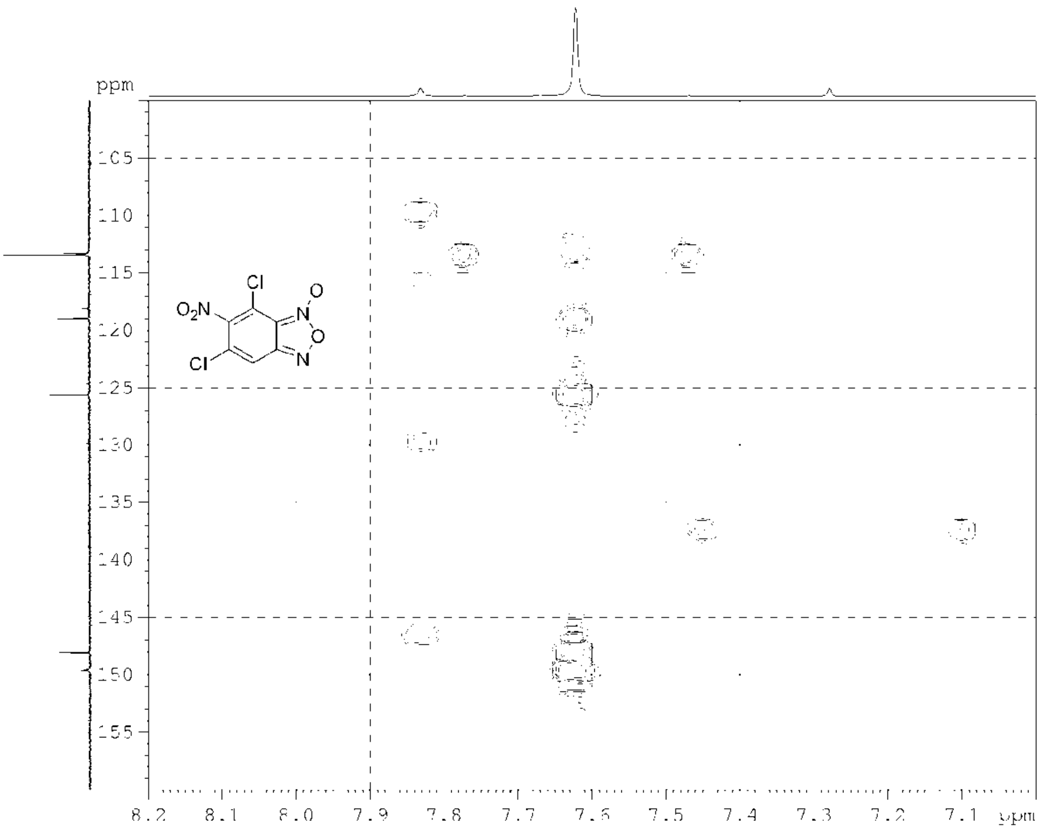

Figure S6. ^{1}H-^{13}C HMBC NMR spectrum of **1**, $CDCl_3$, 253K.

Table S2. Experimental and calculated NMR parameters for **1** (253K)

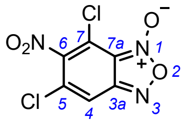 **1A**			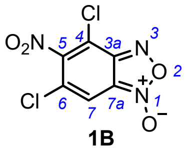 **1B**		
Energy, kCal/mol					
0			1.63		
Nucleus	Experimental	Calculated	Nucleus	Experimental	Calculated
C5	129.9	135.8	C6	125.6	129.6
C4	118.1	116.3	C7	113.5	111.9
C3a	149.5	147.2	C7a	113.3	111.3
C7a	109.6	109.5	C3a	148.1	145.9
C7	115.5	120.9	C4	119.0	123.5
C6	146.6	145.4	C5	149.6	149.1
N3	-*	378.8	N1	-	373.8
N1	-	375.2	N3	-	383.2
NO_2	-	374.0	NO_2	-	373.7
H4	7.83	7.58	H7	7.63	7.43

* - CS was not measured due to low s/n ratio

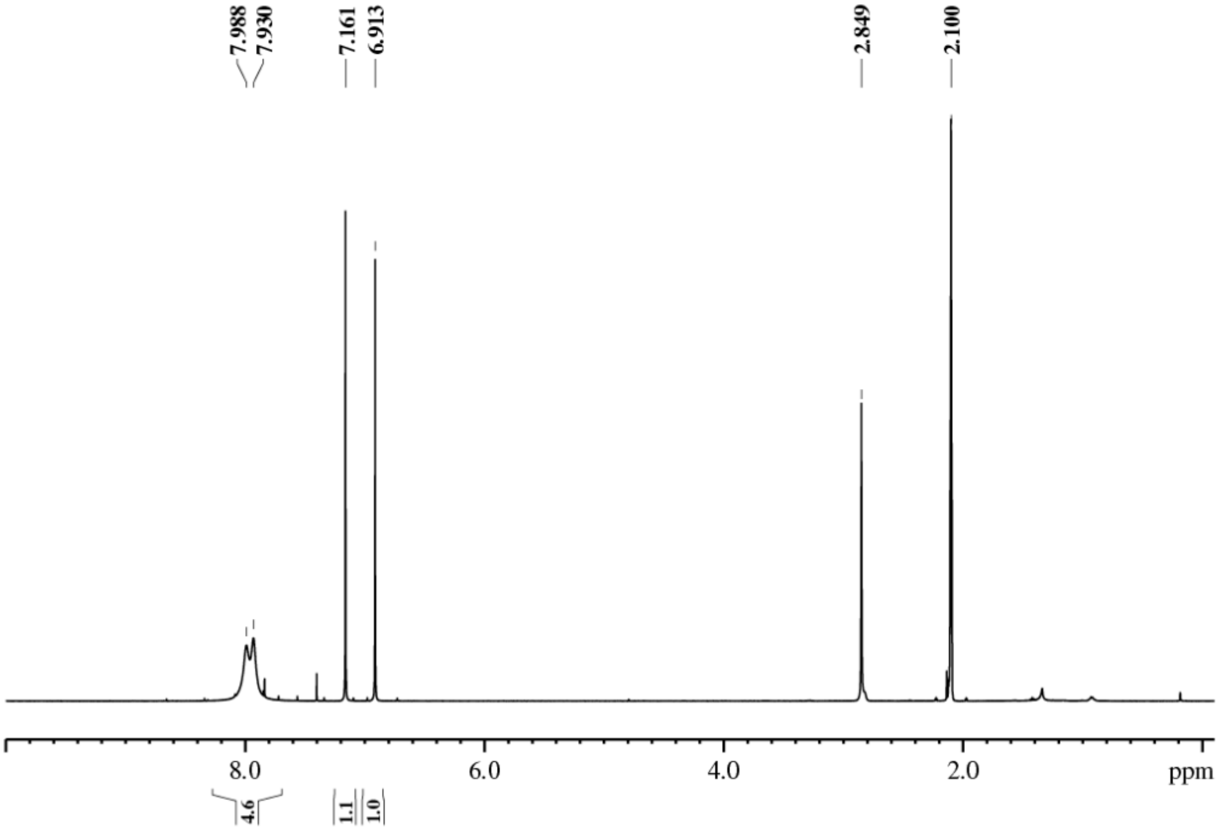

Figure S7. ^{1}H NMR (acetone-d_6, 500 MHz, 303 K) mixture of tautomers **3A** and **3B**.

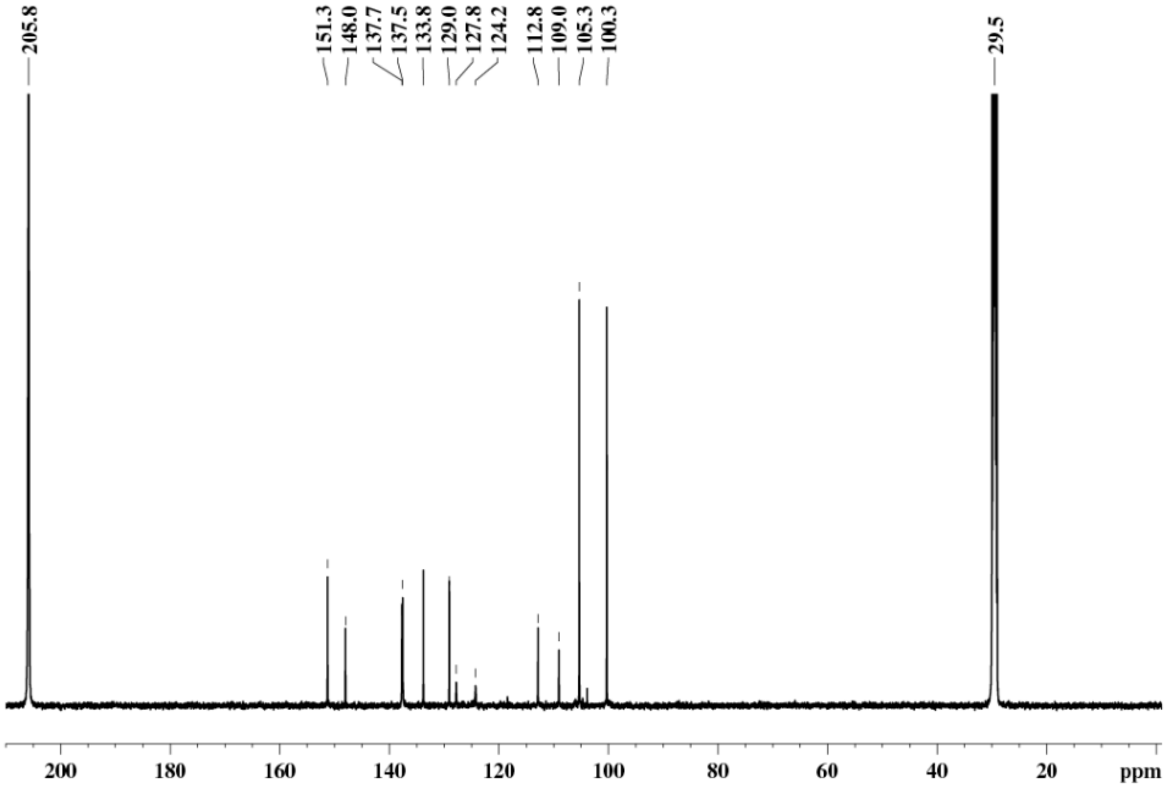

Figure S8. $^{13}C\{1H\}$ NMR (acetone-d_6, 126 MHz, 303 K) mixture of tautomers **3A** and **3B**.

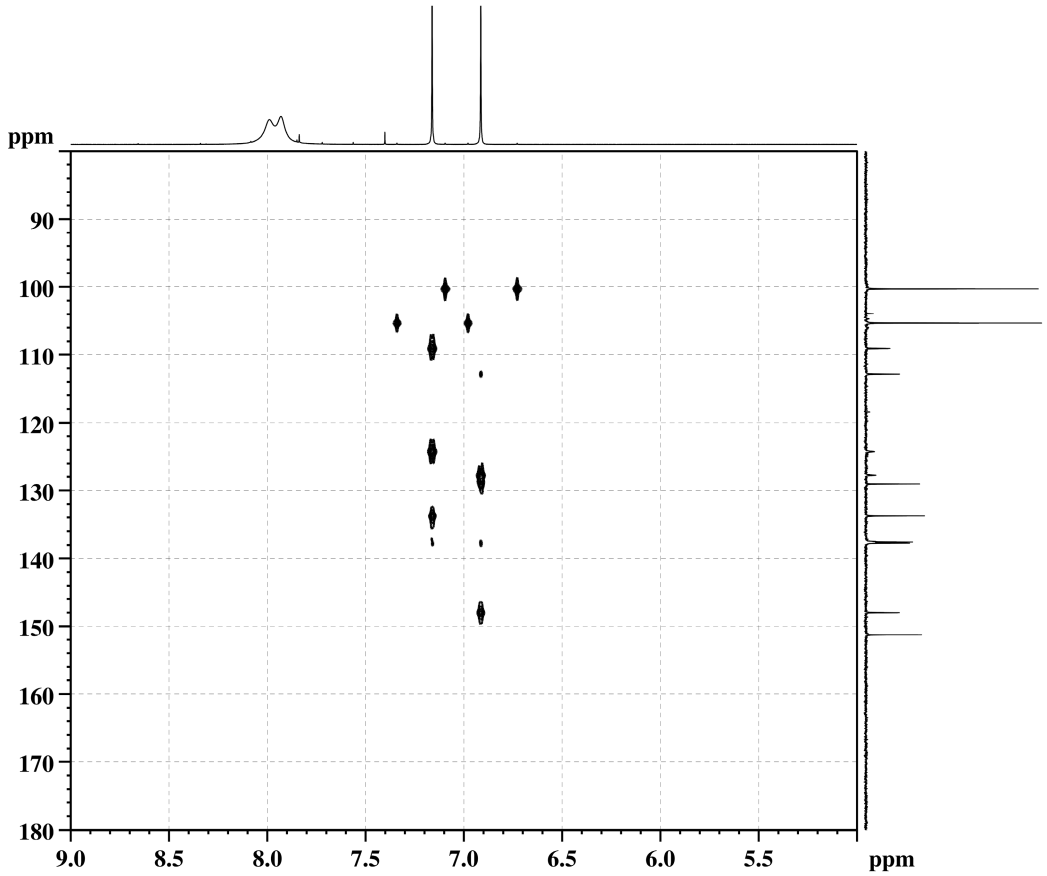

Figure S9. HMBC(^{13}C-^{1}H) NMR (acetone-d_6, 303 K) mixture of tautomers **3A** and **3B**.

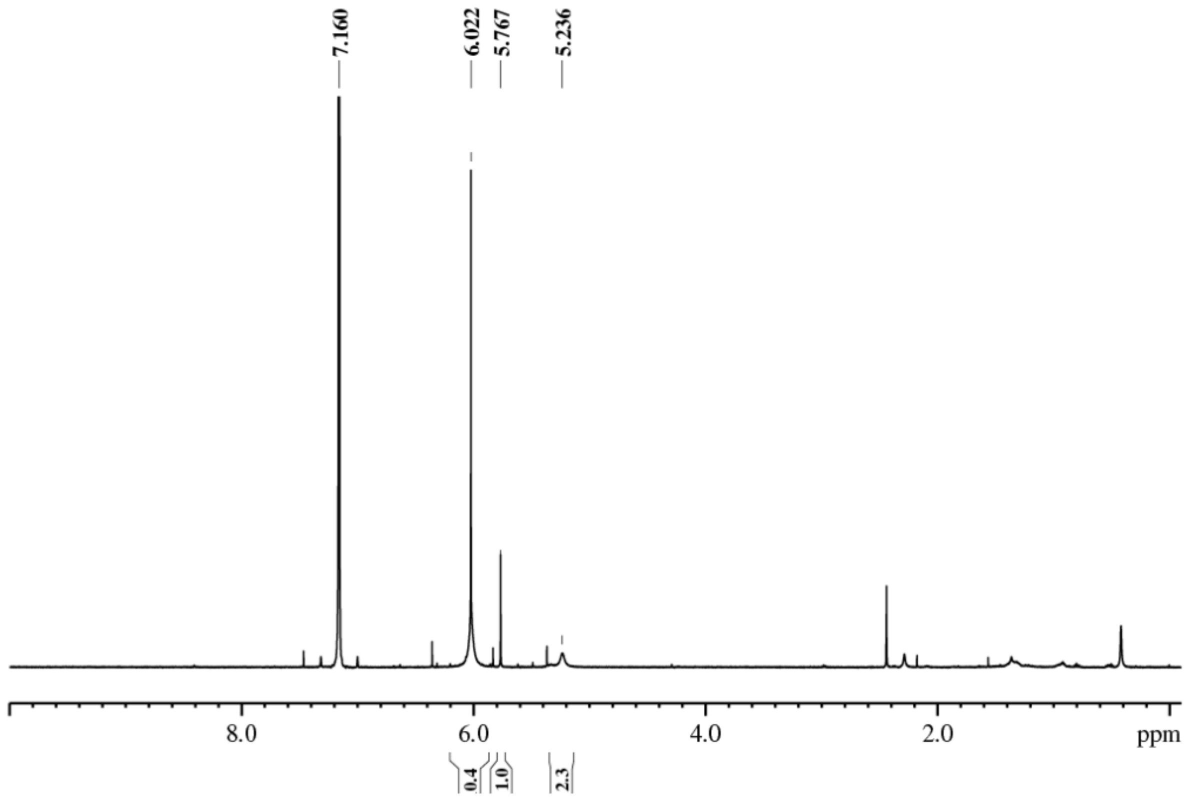

Figure S10. ^{1}H NMR (benzene-d_6, 500 MHz, 303 K) mixture of tautomers **3A** and **3B**.

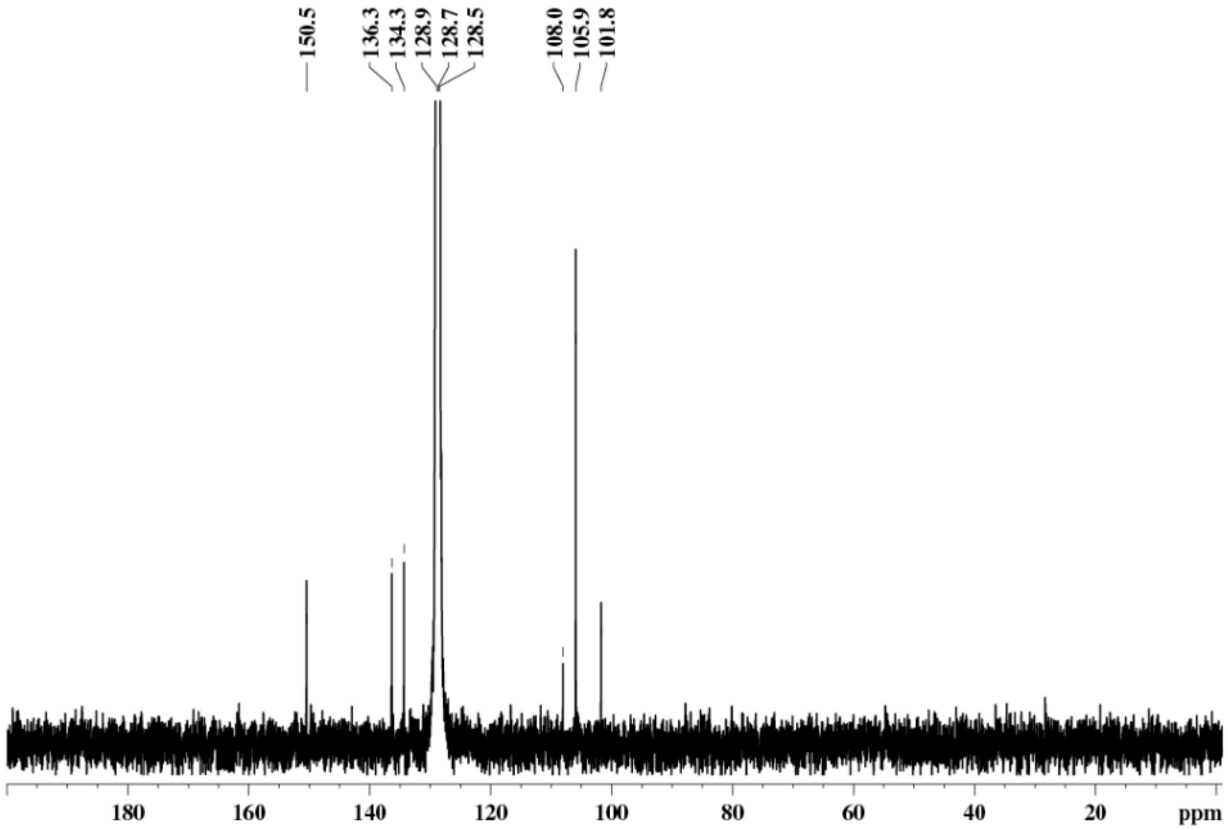

Figure S11. $^{13}C\{1H\}$ NMR (benzene-d_6, 126 MHz, 303 K) mixture of tautomers **3A** and **3B**.

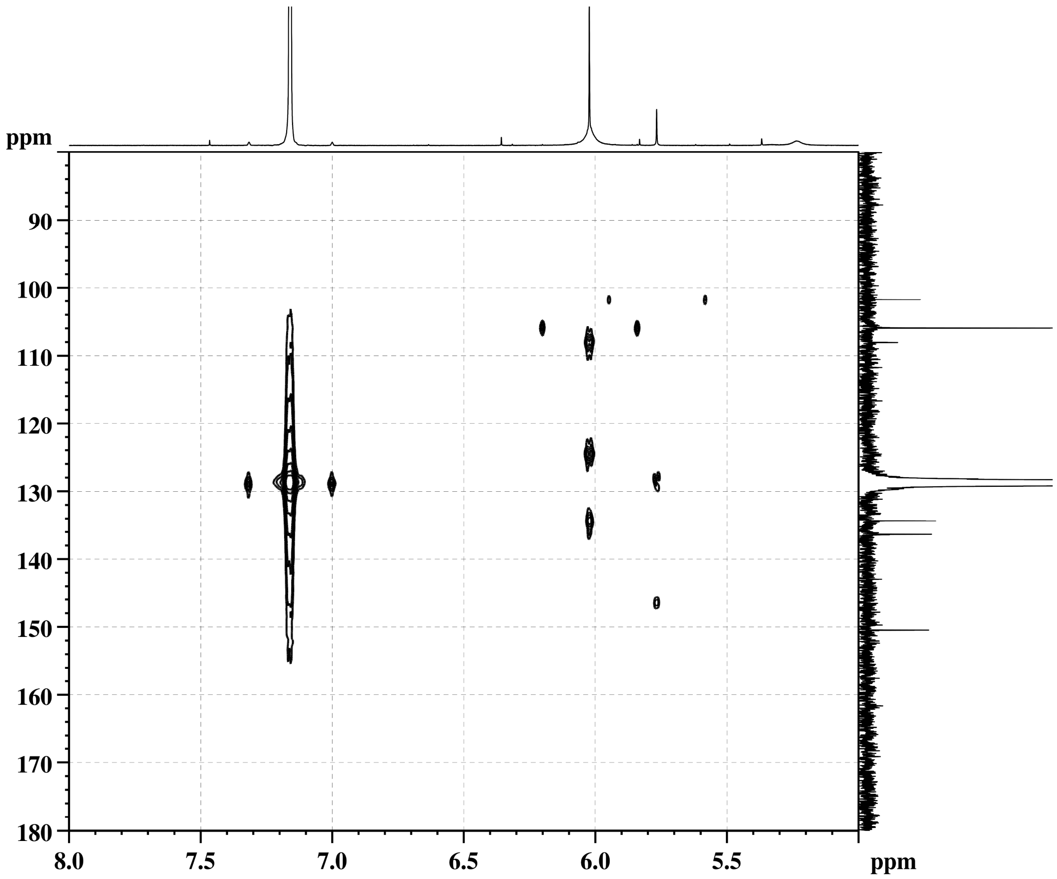

Figure S12. HMBC(^{13}C-^{1}H) NMR (benzene-*d6*, 303 K) mixture of tautomers **3A** and **3B**.

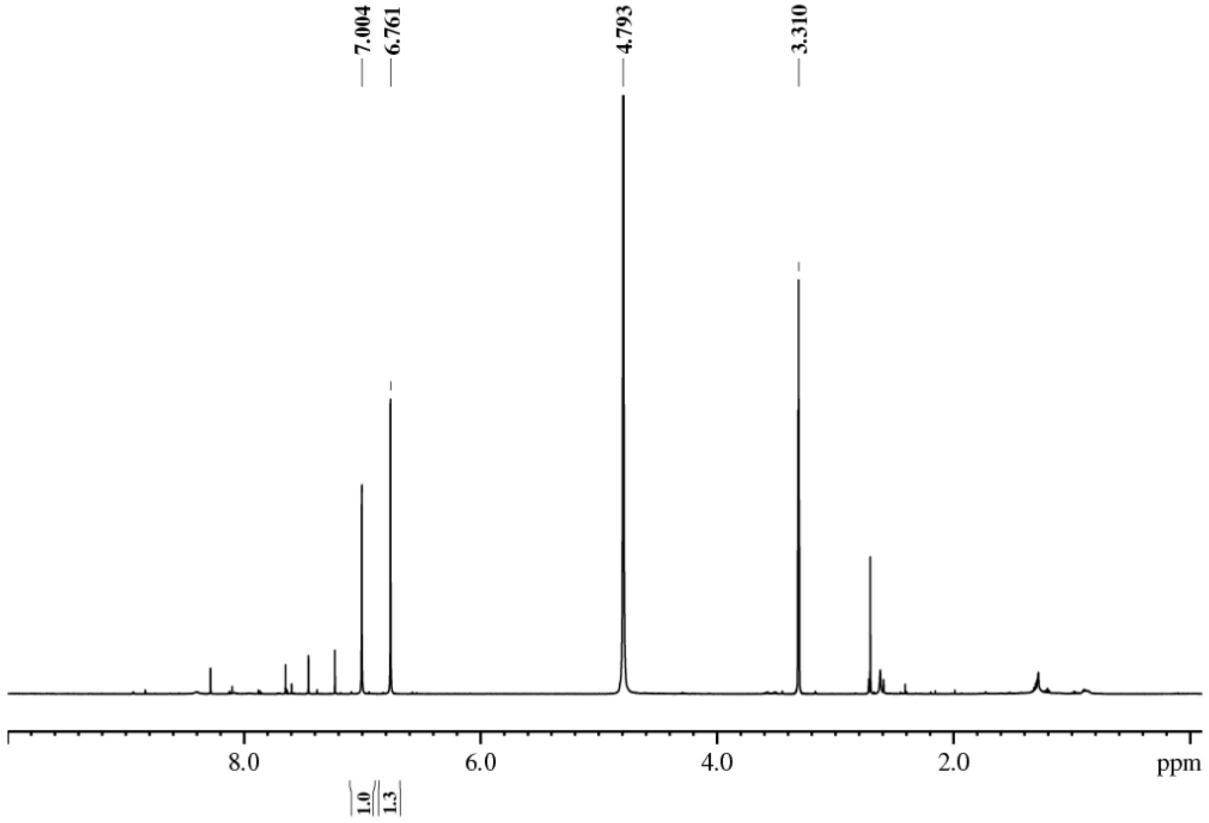

Figure S13. ^{1}H NMR (methanol-*d4*, 500 MHz, 303 K) mixture of tautomers **3A** and **3B**.

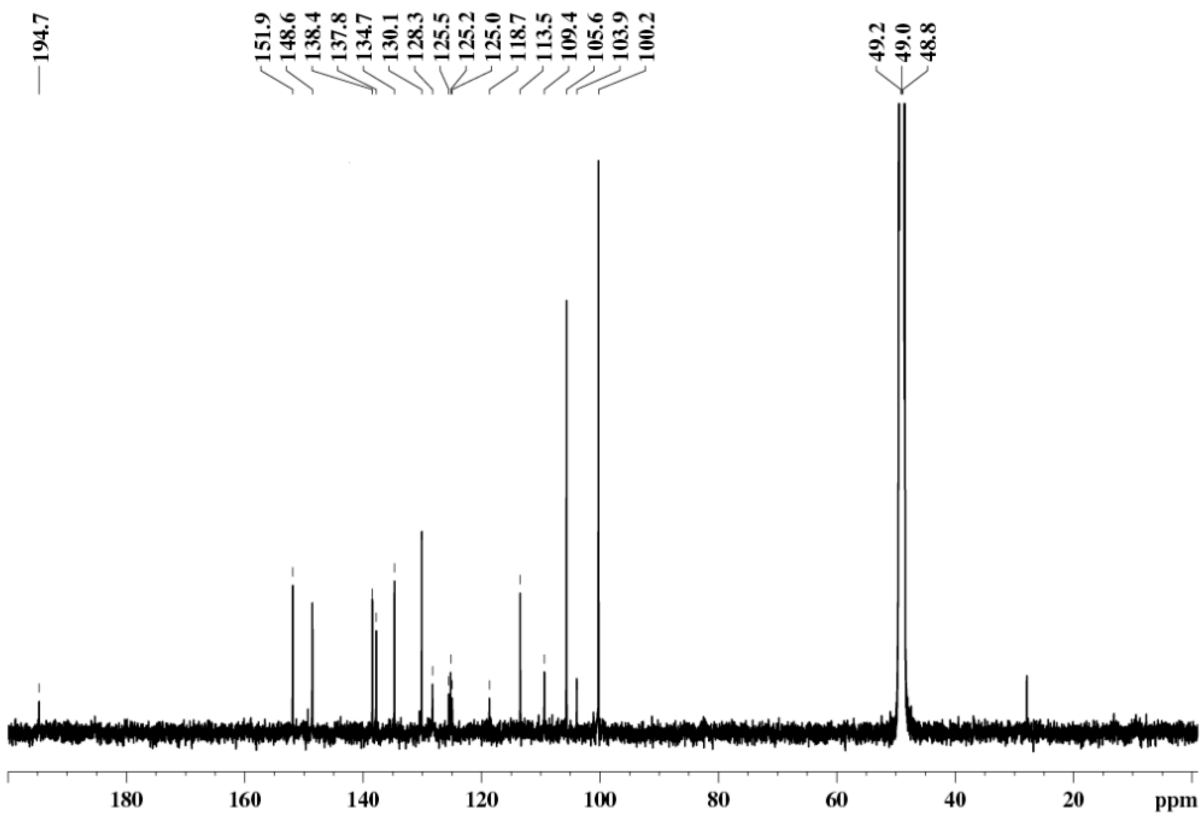

Figure S14. $^{13}C\{^1H\}$ NMR (methanol-d_4, 126 MHz, 303 K) mixture of tautomers **3A** and **3B**.

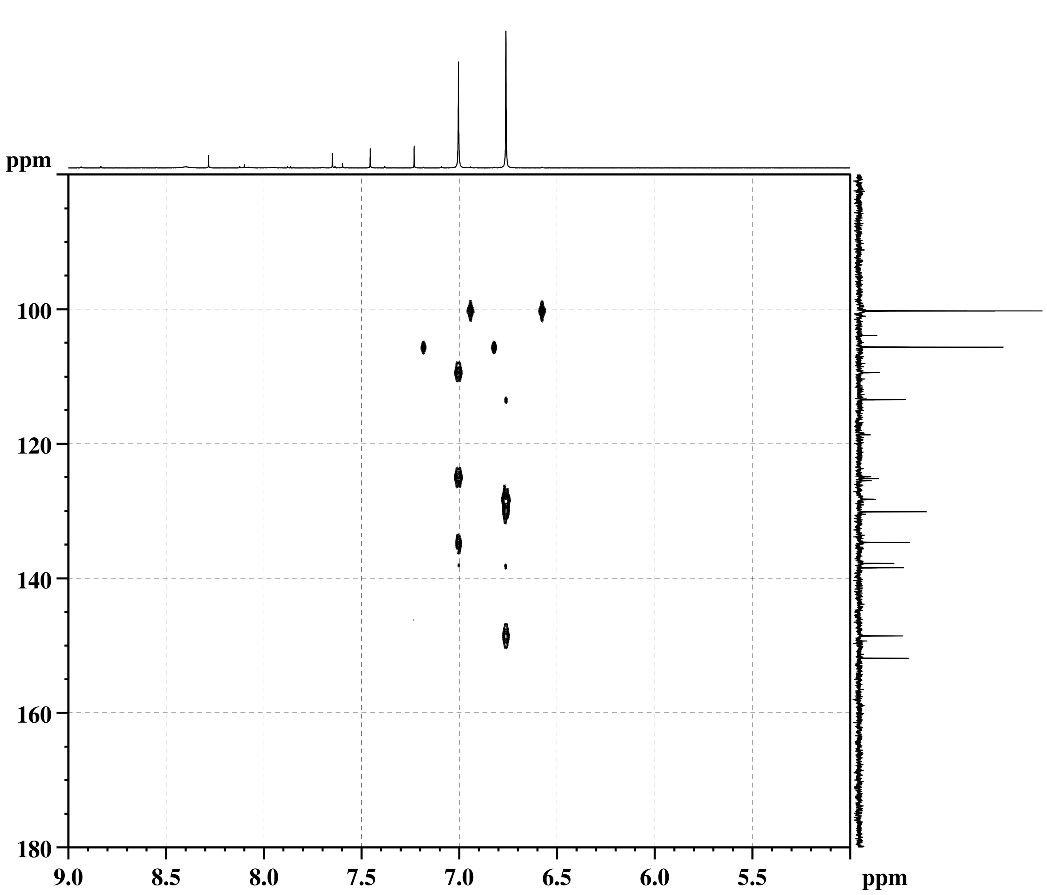

Figure S15. HMBC(^{13}C-^{1}H) NMR (methanol-d_4, 303 K) mixture of tautomers **3A** and **3B**.

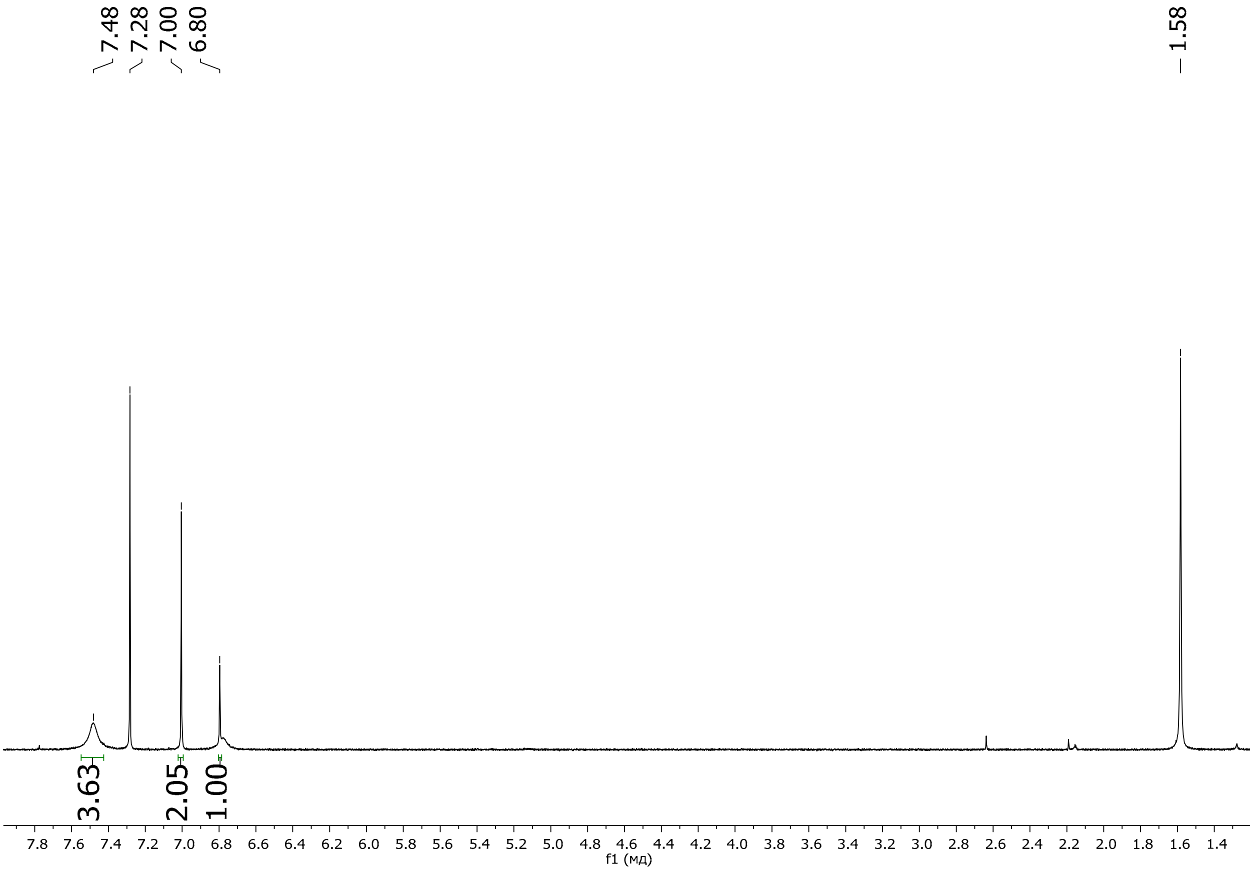

Figure S16. ^{1}H NMR ($CDCl_3$, 500 MHz, 303 K) mixture of tautomers **3A** and **3B**.

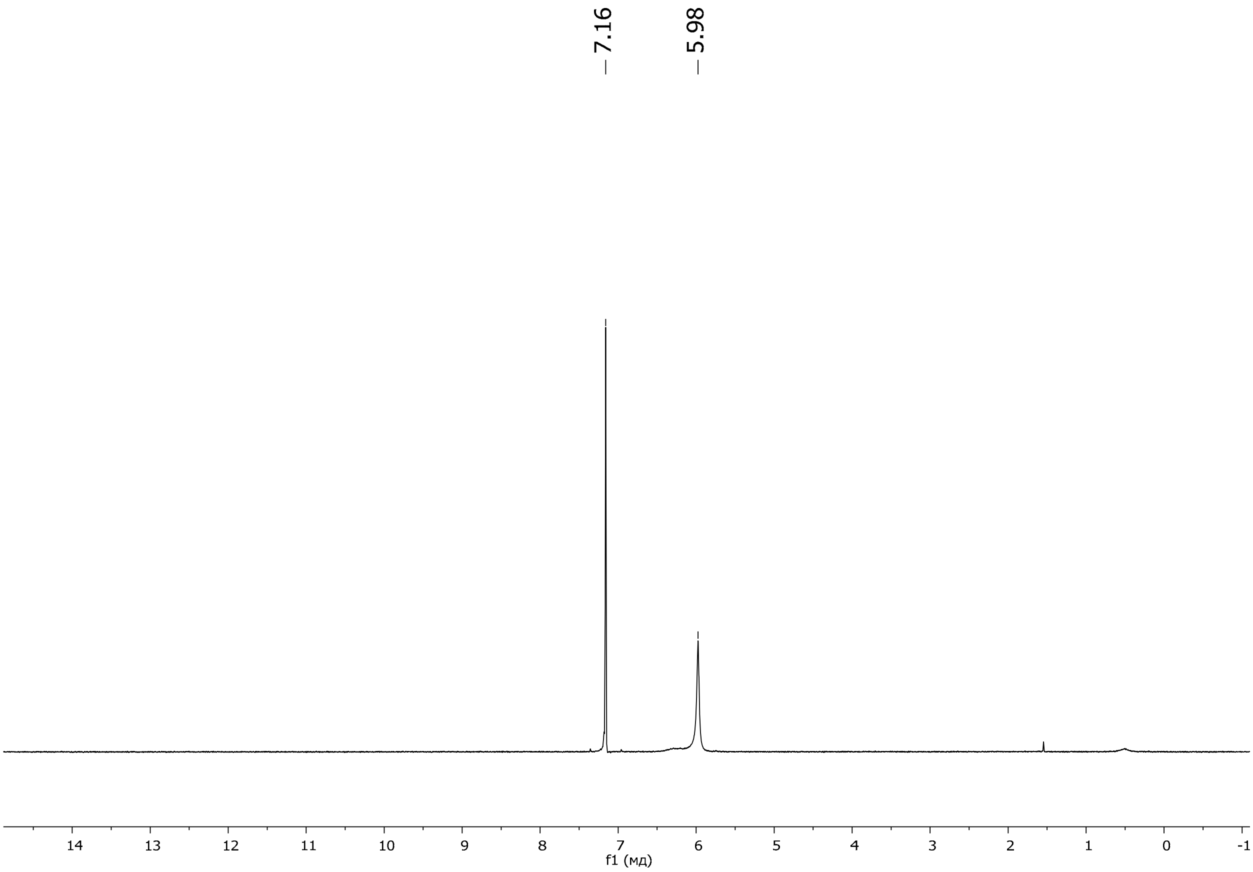

Figure S17. ^{1}H NMR (benzene-d_6, 500 MHz, 303 K) of compound **1**.

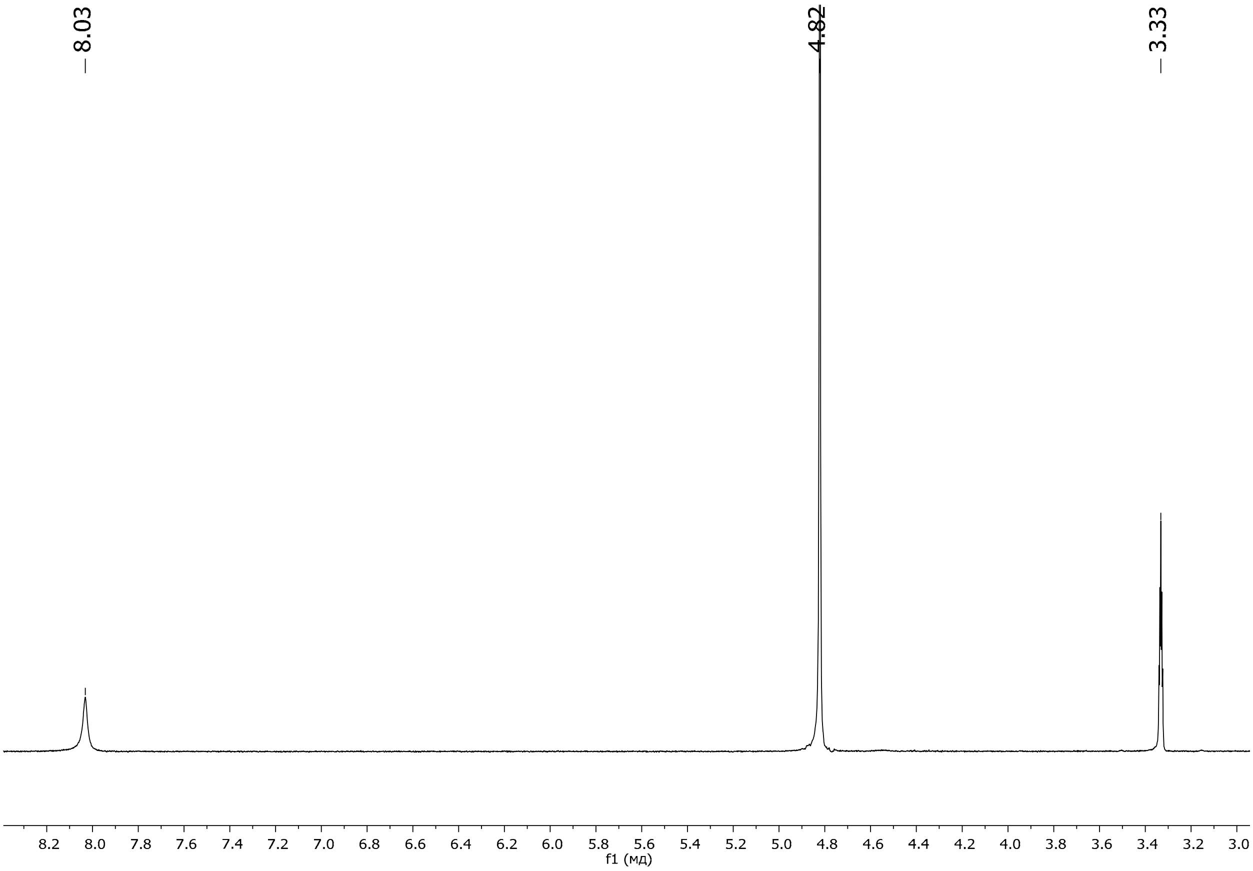

Figure S18. ^{1}H NMR (methanol-d_4, 500 MHz, 303 K) of compound **1**.

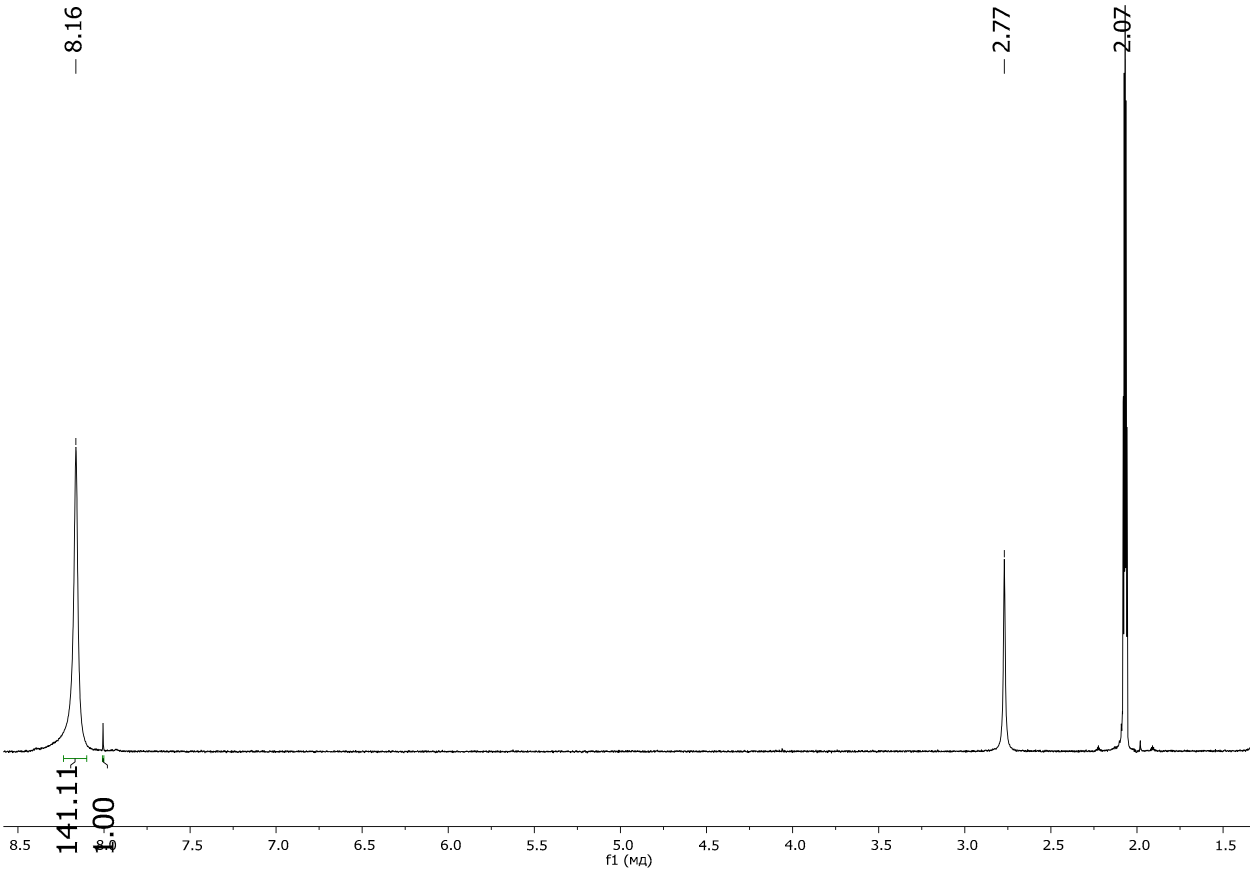

Figure S19. ^{1}H NMR (acetone-d_6, 500 MHz, 303 K) of compound **1**.

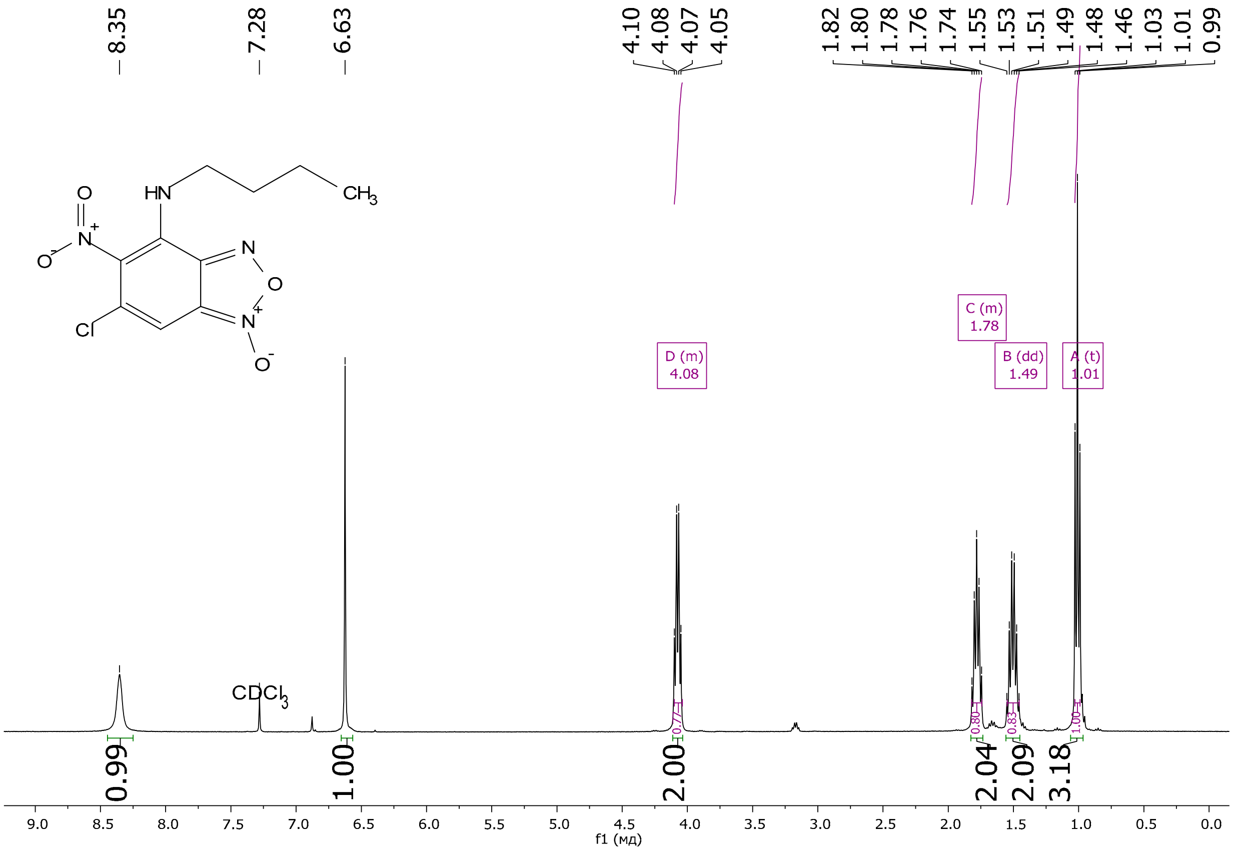

Figure S20. ^{1}H NMR ($CDCl_3$, 500 MHz, 303 K) of compound **5**.

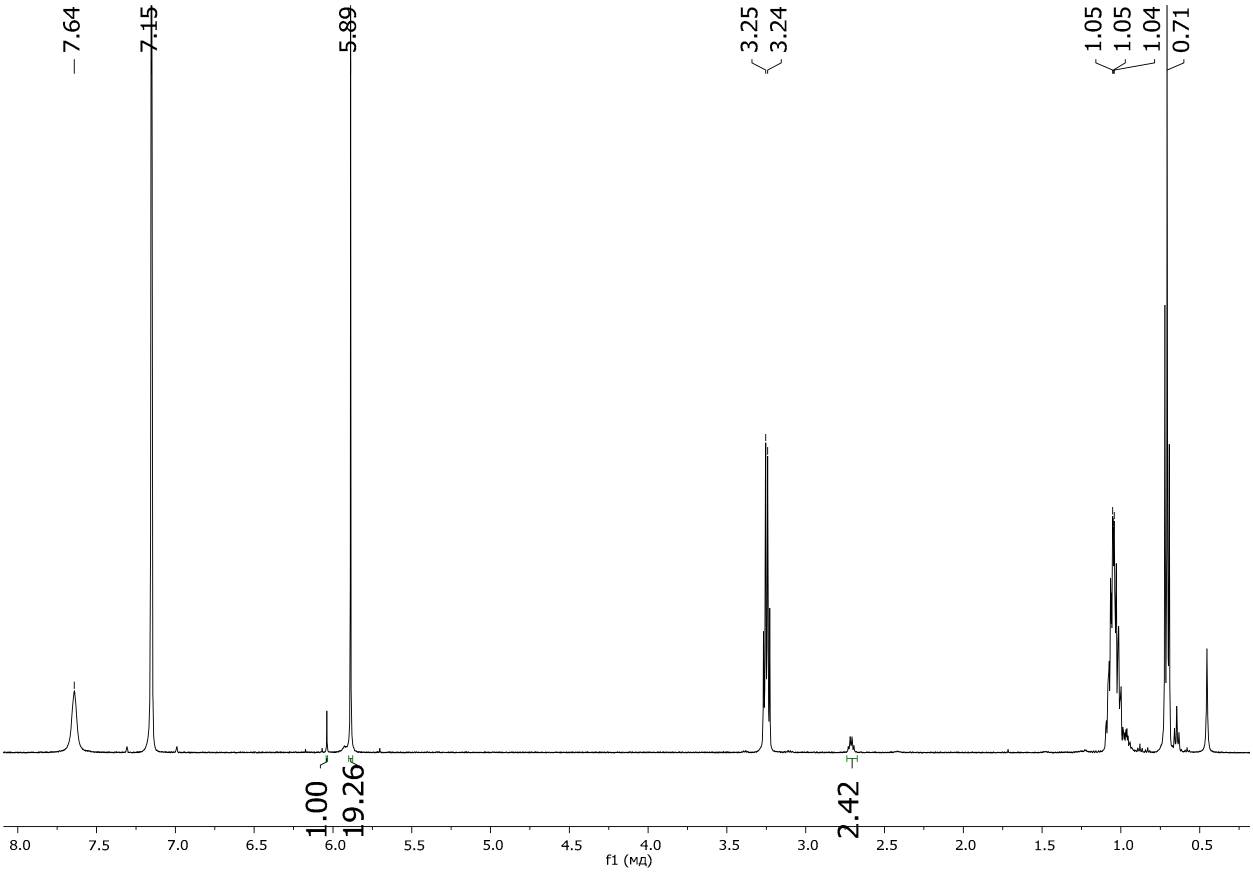

Figure S21. ^{1}H NMR (benzene-d_6, 500 MHz, 303 K) of compound **5**.

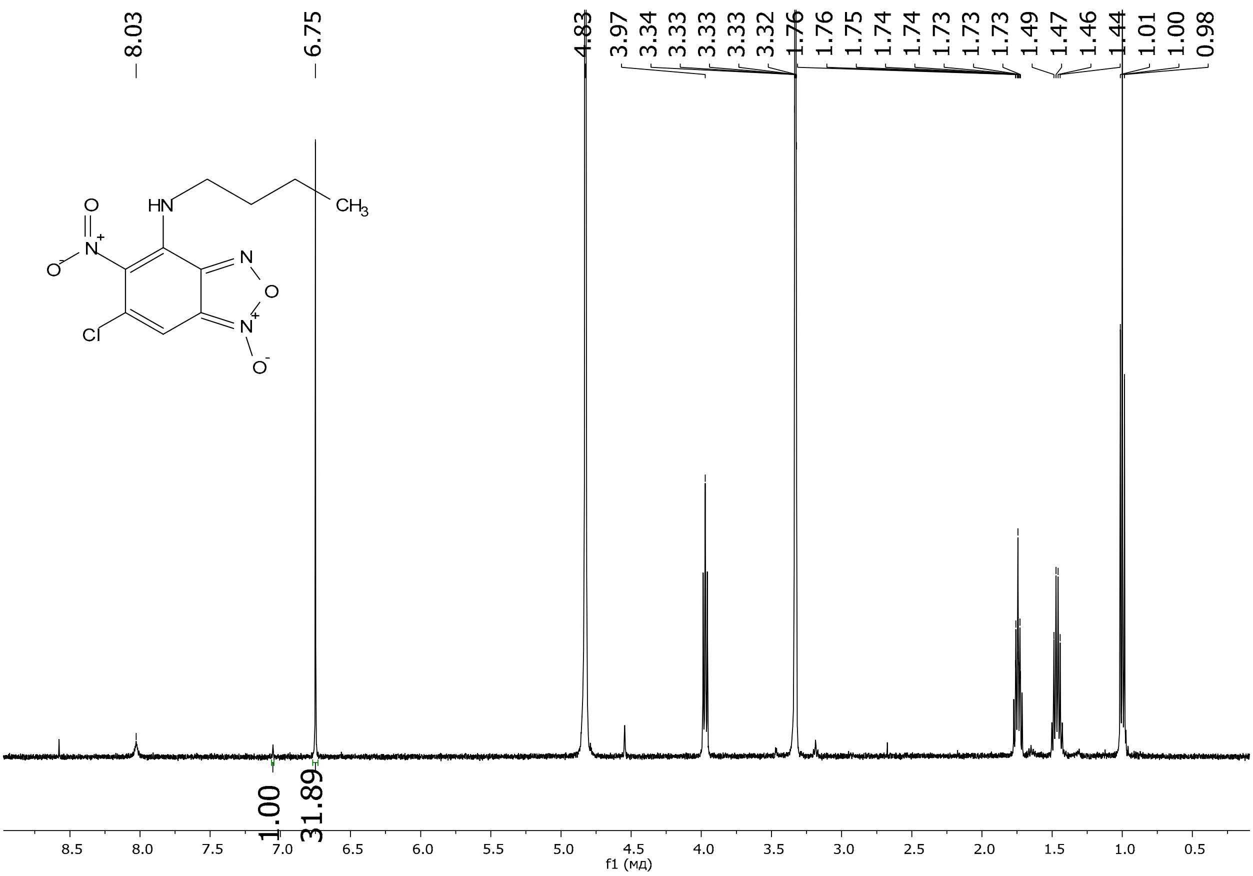

Figure S22. ^{1}H NMR (methanol-*d*$_4$, 500 MHz, 303 K) of compound **5**.

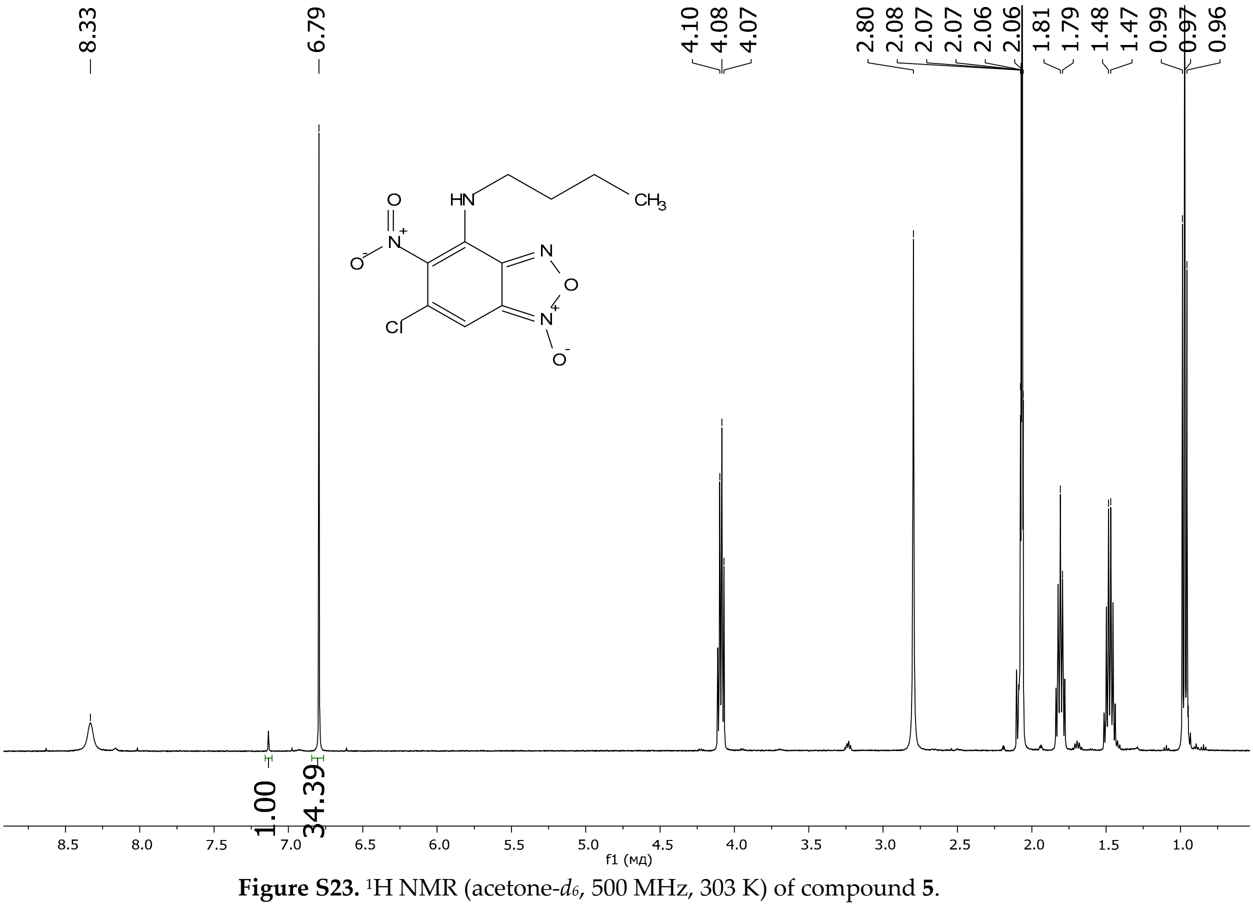

Figure S23. ^{1}H NMR (acetone-*d*$_6$, 500 MHz, 303 K) of compound **5**.

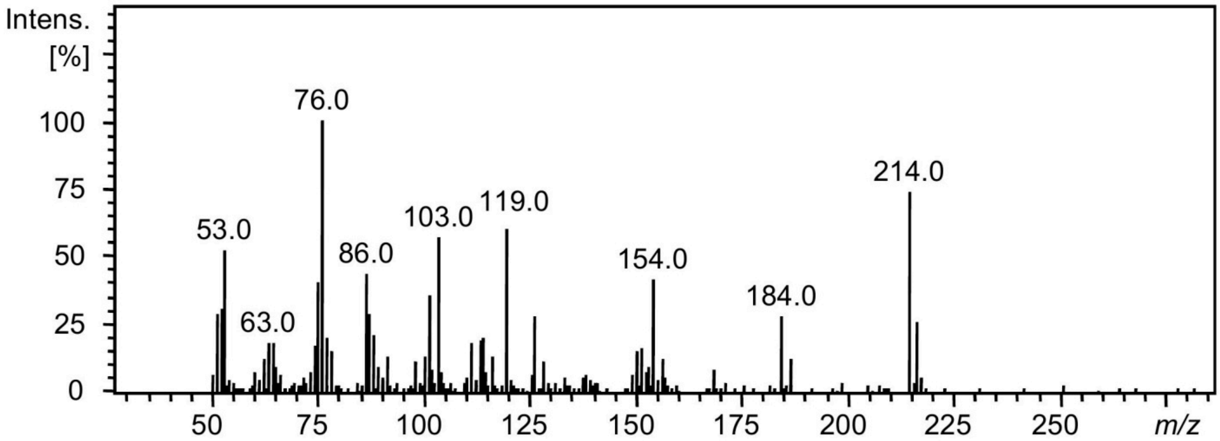

Figure S24. EI mass spectrum (70 eV) of compound **3**

Table S3. Change in chemical shifts between tautomers and when changing the solvent for compound **3A/3B**.

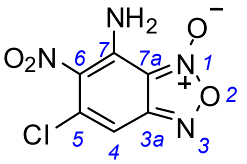

3A

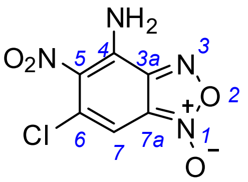

3B

**3A**						**3B**								
	acetone-d_6	C_6D_6		MeOD			acetone-d_6		C_6D_6			MeOD		
			Δ1		Δ1			Δ2		Δ2	Δ1		Δ2	Δ1
**(C7)**	151.3	150.5	*-0.8*	151.9	*0.6*	**(C4)**	148	*-3.3*	146.5	*-4.0*	*-1.5*	148.6	*-3.3*	*0.6*
**(C5)**	137.6	136.3	*-1.3*	137.8	*0.2*	**(C6)**	137.7	*0.1*	n/o/			138.4	*0.6*	*0.7*
**(C3a)**	133.8	134.3	*0.5*	134.7	*0.9*	**(C7a)**	112.8	*-21.0*	n/o/			113.5	*-21.2*	*0.7*
**(C6)**	124.2	124.6	*0.4*	125.0	*0.8*	**(C5)**	127.8	*3.6*	127.7	*3.1*	*-0.1*	128.3	*3.3*	*0.5*
**(C7a)**	109.0	108	*-1.0*	109.4	*0.4*	**(C3a)**	129.0	*20.0*	129.3	*21.3*	*0.3*	130.1	*20.7*	*1.1*
**(C4)**	105.3	105.6	*0.3*	105.6	*0.3*	**(C7)**	100.3	*-5.0*	101.8	*-3.8*	*1.5*	100.2	*-5.4*	*-0.1*

Δ1=δ(in solvent)- δ (in acetone-d_6); Δ2= δ(3B)- δ(3A)
